# Design, Synthesis and Structure-Activity Relationship Studies of Novel Survivin Inhibitors with Potent Anti-Proliferative Properties

**DOI:** 10.1371/journal.pone.0129807

**Published:** 2015-06-12

**Authors:** Min Xiao, Jin Wang, Zongtao Lin, Yan Lu, Zhenmei Li, Stephen W. White, Duane D. Miller, Wei Li

**Affiliations:** 1 Department of Pharmaceutical Sciences, University of Tennessee Health Science Center, Memphis, Tennessee, United States of America; 2 Department of Structure Biology, St. Jude Children’s Research Hospital, Memphis, Tennessee, United States of America; Medical University of Gdańsk, POLAND

## Abstract

The anti-apoptotic protein survivin is highly expressed in most human cancer cells, but has very low expression in normal differentiated cells. Thus survivin is considered as an attractive cancer drug target. Herein we report the design and synthesis of a series of novel survivin inhibitors based on the oxyquinoline scaffold from our recently identified hit compound UC-112. These new analogs were tested against a panel of cancer cell lines including one with multidrug-resistant phenotype. Eight of these new UC-112 analogs showed IC_50_ values in the nanomole range in anti-proliferative assays. The best three compounds among them along with UC-112 were submitted for NCI-60 cancer cell line screening. The results indicated that structural modification from UC-112 to our best compound **4g** has improved activity by four folds (2.2 μM for UC-112 vs. 0.5 μM for **4g**, average GI_50_ values over all cancer cell lines in the NCI-60 panel).Western blot analyses demonstrated the new compounds maintained high selectivity for survivin inhibition over other members in the inhibition of apoptosis protein family. When tested in an A375 human melanoma xenograft model, the most active compound **4g** effectively suppressed tumor growth and strongly induced cancer cell apoptosis in tumor tissues. This novel scaffold is promising for the development of selective survivin inhibitors as potential anticancer agents.

## Introduction

Survivin is a unique member of inhibitor of apoptosis protein (IAP) family.[1] It is overexpressed in most human cancer cells, but is rarely expressed in adult differentiated tissues.[2–5] This attribute distinguishes survivin from other IAPs which are usually expressed in both cancer and normal cells. Survivin promotes cell proliferation and inhibits apoptosis,[3, 6–9] facilitates angiogenesis in tumors,[10–12] and its expression has been shown to strongly correlate with multiple mechanisms of drug resistance.[13–15] Therefore, survivin is widely considered to be an ideal cancer drug target. Several molecules in different categories including antisense oligonucleotides, dominant-negative mutants, ribozymes, small interfering RNAs, cancer vaccine and small molecules have been identified as survivin inhibitors.[16] However, due to the challenging requirement to efficiently disrupt protein-protein interactions, the pool of existing small molecule survivin inhibitors is quite small.[17] The efficacy of those survivin inhibitors is also limited.[16] For example, the reported clinical candidate, small molecule survivin inhibitor YM155 has been shown to be a substrate of P-glycoprotein (Pgp) drug efflux pump, which suggests that YM155 has limited efficacy in multiple drug resistant phenotypes.[18] Therefore, it is highly significant to develop new survivin inhibitors that can overcome multidrug resistance, which is an important goal of our research.

**Fig 1:**
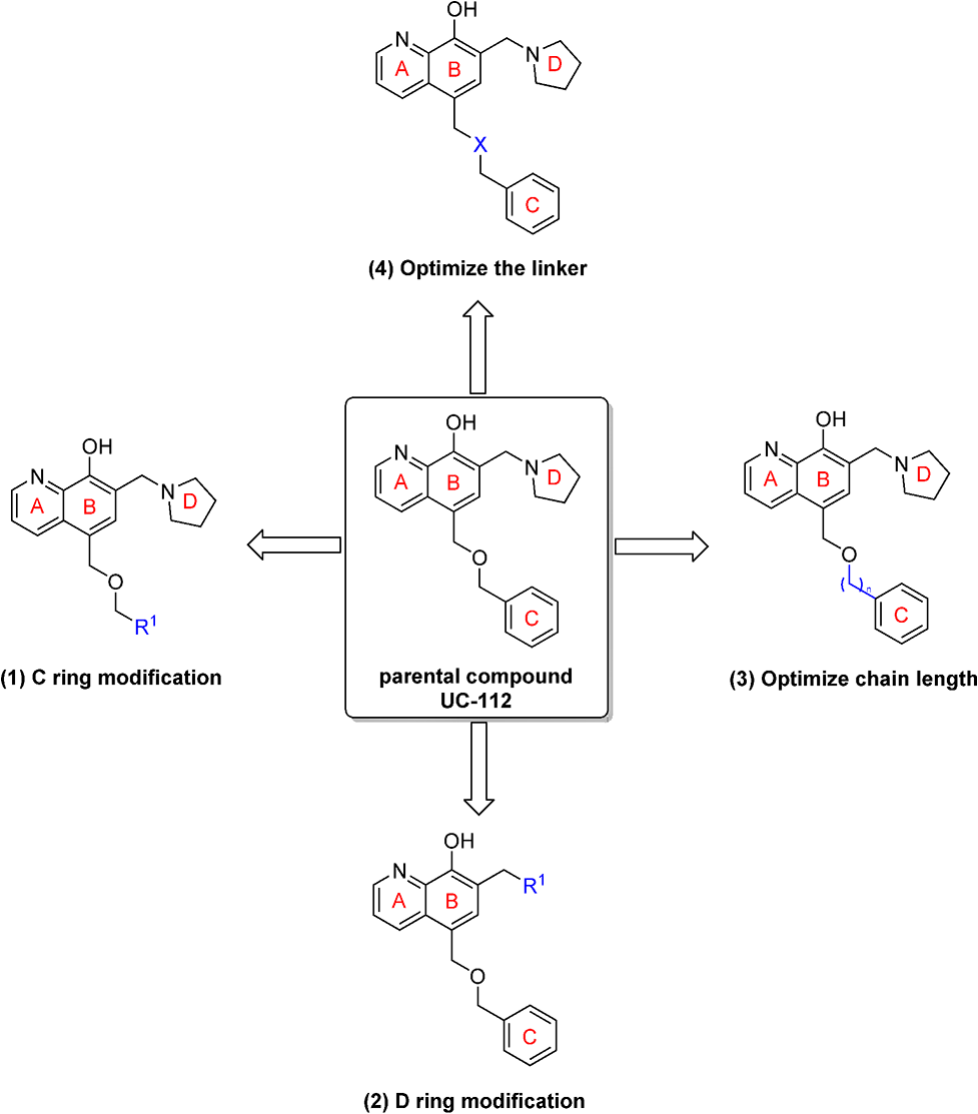
Targeted modification to design new UC-112 analogs.

We recently discovered that UC-112, [5-((benzyloxy)methyl)-7-(pyrrolidin-1-ylmethyl)quinolin-8-ol], is a potent, selective survivin inhibitor (Fig 1).[19] UC-112 inhibits tumor cell growth in several cancer cell lines *in vitro* and suppresses melanoma tumor growth *in vivo*. [19] Mechanistic studies indicated that UC-112 selectively inhibits survivin expression and induces strong cancer cell apoptosis. In order to further optimize this unique oxyquinoline scaffold and explore its structure-activity relationships (SAR), we designed several focused sets of new UC-112 analogs (Fig 1). We introduced four major modifications to the structure of UC-112. First, we modified the C ring moiety by introducing substitutions to the phenyl ring or replacing it with other groups. Secondly, we modified the D ring moiety with other cyclic amines. We also explored different chain lengths between the oxygen linker and the C ring moiety. Finally, the linker between the B ring and the C ring was modified. By performing these targeted modifications, a series of novel UC-112 analogs were obtained. Biological evaluation of these new analogs revealed their excellent anti-proliferative activity against several cancer cell lines including multidrug-resistant phenotypes. Mechanism of action studies confirmed that these new UC-112 analogs maintained their mechanisms of actions by selectively downregulating the level of survivin among other IAP family proteins. Preliminary *in vivo* evaluation for the most active compound **4g** demonstrated its efficacy against human melanoma tumor growth.

## Results and Discussion

### Chemistry

**Fig 2:**
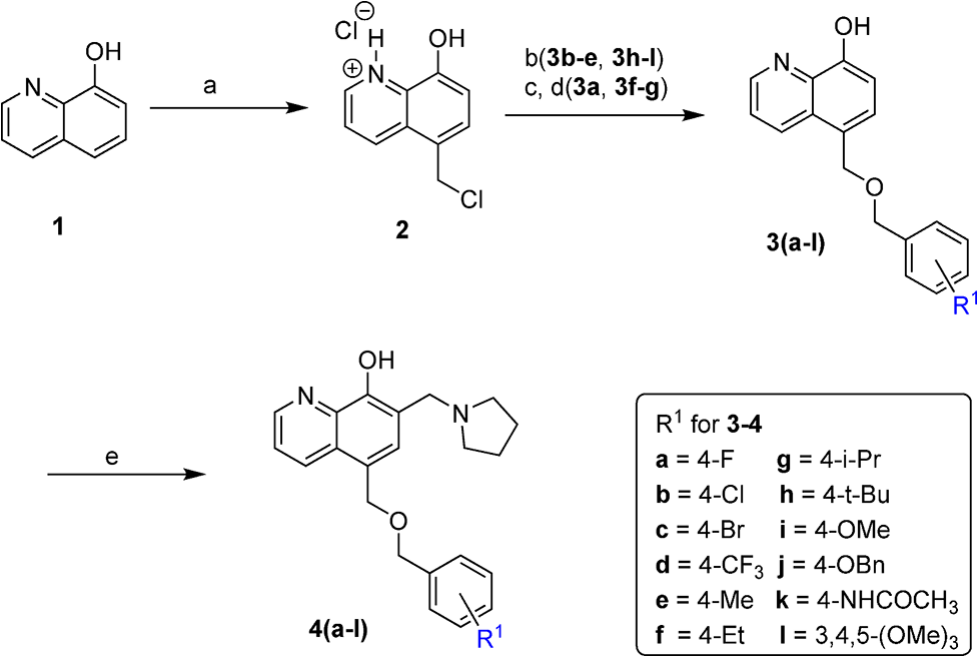
Synthesis of compounds 4a-4l. Reagents and conditions: (a) conc. HCl, ZnCl_2_, HCHO(37% in H_2_O); (b) substituted benzyl alcohol, NaH, THF, reflux; (c) substituted benzyl alcohol, 110 ^o^C; (d) NH_4_OH, H_2_O, Et_2_O, pH 8–10; (e) paraformaldehyde, pyrrolidine,EtOH, reflux.

**Fig 3:**
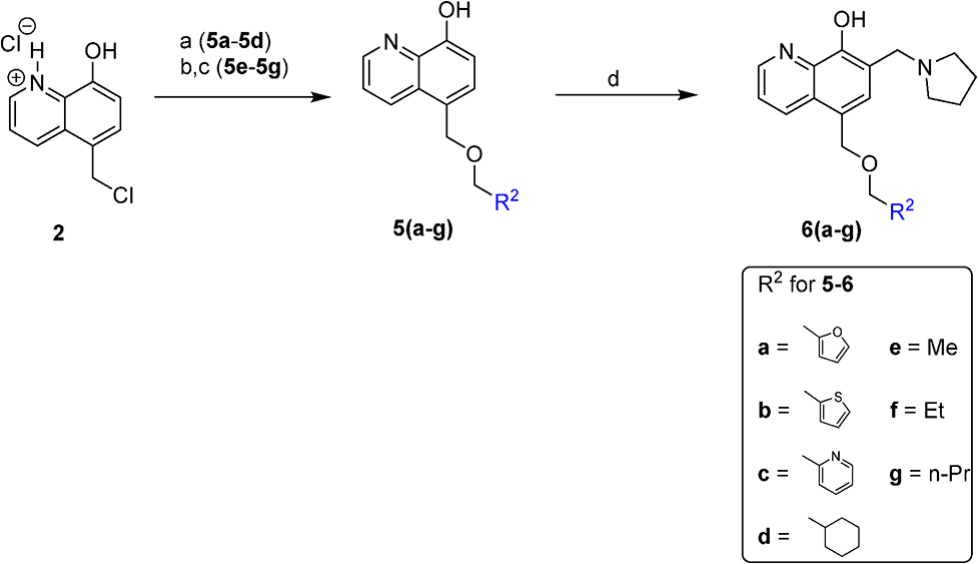
Synthesis of compounds 6a-6g. Reagents and conditions: (a) R^2^CH_2_OH, NaH, THF, reflux; (b) R^2^CH_2_OH, 110 ^o^C; (c) NH_4_OH, H_2_O, Et_2_O, pH 8–10; (d) paraformaldehyde, pyrrolidine,EtOH, reflux.

**Fig 4:**
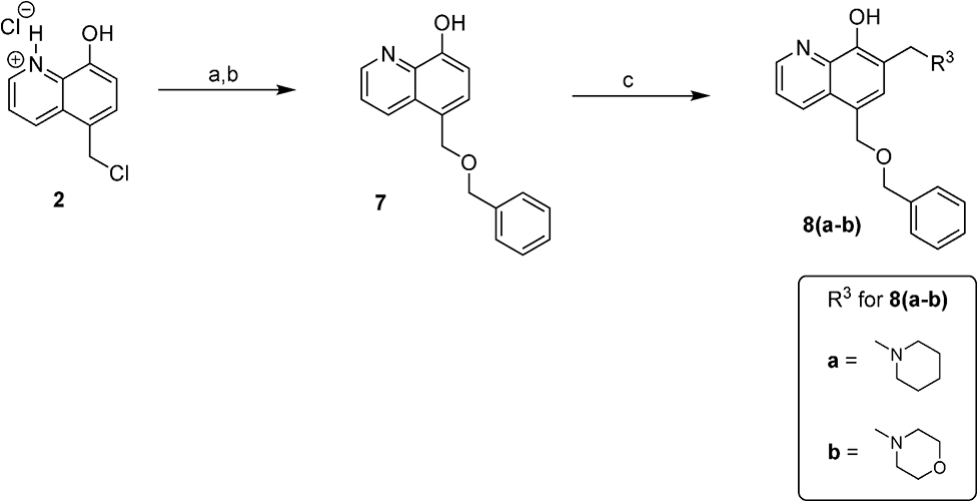
Synthesis of compounds 8a-8b. Reagents and conditions: (a) benzyl alcohol, 110 ^o^C; (b) NH_4_OH, H_2_O, Et_2_O, pH 8–10; (c) paraformaldehyde, piperidine for 8a, morpholine for 8b,EtOH, reflux.

**Fig 5:**
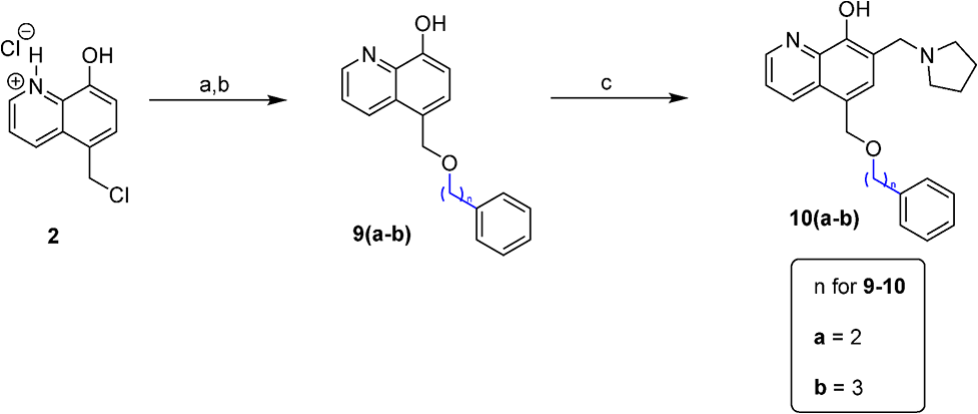
Synthesis of compounds 10a-10b. Reagents and conditions: (a) 2-phenylethanol for 9a, 3-phenyl-1-propanol for 9b, 110 ^o^C; (b) NH_4_OH, H_2_O, Et_2_O, pH 8–10; (c) paraformaldehyde, pyrrolidine, EtOH, reflux.

**Fig 6:**
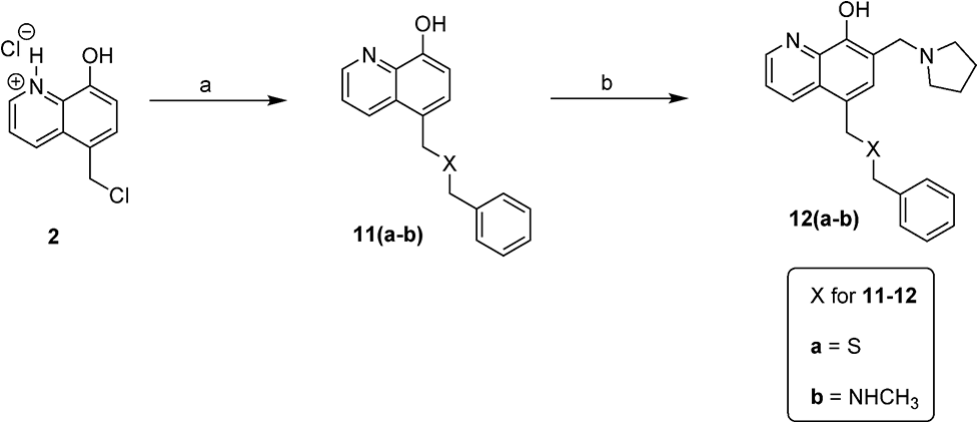
Synthesis of compounds 12a-12b. Reagents and conditions: (a) benzyl mercaptan for **11a**, N-Benzylmethylamine for **11b**, NaH, THF, reflux; (b) paraformaldehyde, pyrrolidine,EtOH, reflux.

The general synthesis of C ring substituted UC-112 analogs (**4a-4l**) is outlined in Fig 2. First 8-hydroxyquinoline reacted with formaldehyde and catalytic zinc chloride in concentrated hydrochloric acid to generate salt **2**.[20] This step introduced a chloromethyl group to the 5-position of quinoline ring. Then salt **2** was allowed to react with different substituted benzyl alcohols to form ethers **3a-3l**, by two different methods. Ethers **3b-3e** and ethers **3h-3l** were synthesized using step b in which substituted benzyl alcohols were allowed to react with salt **2** in the presence of sodium hydride in anhydrous THF. Ethers **3a**, **3f** and **3g** were synthesized through step c and step d. In step c, substituted benzyl alcohols reacted directly with salt **2** with heating to form different salts which were converted to free base by adjusting pH with NH_4_OH solution in step c.[21] The synthesized ethers were than submitted to Mannich reaction conditions with paraformaldehyde and pyrrolidine in ethanol to form the final compounds **4a** to **4l**.[22] Compounds **6a-6g** with the C ring moiety in UC-112 replaced by different function groups were prepared as Fig 3 shown. The ethers **5a-5g** were first synthesized using similar approach as shown in Fig 2. Then these ethers were converted to **6a-6g** *via* the Mannich reaction. The synthesis of D ring modified UC-112 analogs **8a** and **8b** is shown in Fig 4. Salt **2** first reacted with benzyl alcohol to form intermediate **7**, which was allowed to react with piperidine or morpholine *via* the Mannich reaction to form compounds **8a** and **8b.** Compounds **10a** and **10b** which have different chain lengths between oxygen and the phenyl ring from parent compound UC-112 were made using the method shown in Fig 5. First salt **2** reacted with 2-phenylethanol and 3-phenyl-1-propanol respectively to form **9a** and **9b**, which then underwent the Mannich reaction with paraformaldehyde and pyrrolidine to form compounds **10a** and **10b**. Finally, compounds **12a** and **12b,** which have different linkers between the B ring and C ring as compared to the parent compound were made (Fig 6). First salt **2** reacted with benzyl mercaptan or N-benzymethylamine to form intermediate **11a** and **11b**. Then **11a** and **11b** underwent the Mannich reaction to form compounds **12a** and **12b**.

### Biological results and discussion

All UC-112 analogs were evaluated for their cytotoxicity in a panel of human cancer cell lines including melanoma (A375, M14 and its multidrug resistant daughter line M14/LCC6MDR1) and prostate cancer (PC-3). UC-112 was also included in the assays, serving as positive control and basis for comparison. These *in vitro* biological results are summarized in Tables 1–4 and discussed in detail in the following sections.

**Table 1:** *In vitro* growth inhibitory effects of UC-112 analogs with C ring substitutions.

**ID**	**IC_50_ values ± SEM (μM)**				
	**A375**	**M14**	**M14/LCC6MDR1**	**PC-3**	**Resistance Index**
**4a**	1.5 ± 0.2	1.8 ± 0.3	4.4 ± 0.7	0.7 ± 0.2	2.4
**4b**	1.5 ± 0.3	1.1 ± 0.1	2.4 ± 0.5	0.9 ± 0.1	2.2
**4c**	0.9 ± 0.2	1.0 ± 0.2	1.8 ± 0.6	0.7 ± 0.3	1.8
**4d**	0.9 ± 0.3	1.0 ± 0.4	1.9 ± 0.5	1.0 ± 0.1	1.9
**4e**	1.7 ± 0.3	2.3 ± 0.5	4.1 ± 2.0	1.1 ± 0.2	1.8
**4f**	0.9 ± 0.1	0.9 ± 0.1	2.7 ± 1.0	0.9 ± 0.1	3.0
**4g**	0.9 ± 0.1	0.8 ± 0.2	1.8 ± 0.4	0.7 ± 0.1	2.3
**4h**	1.1 ± 0.4	ND^a^	1.5 ± 0.7	1.5 ± 0.3	ND
**4i**	1.8 ± 0.2	1.5 ± 0.4	3.8 ± 0.6	1.0 ± 0.4	2.5
**4j**	2.8 ± 0.1	ND	12.1 ± 0.5	ND	ND
**4k**	4.7 ± 0.8	ND	5.5 ± 1.3	ND	ND
**4l**	2.8 ± 0.4	3.6 ± 1.0	>30	1.6 ± 0.3	> 8.3
**UC-112**	1.9 ± 0.6	2.1 ± 0.3	3.2 ± 0.5	1.6 ± 1.0	1.5
**YM-155**	ND	0.003	>10	ND	>2941

a ND: Not Determined. Resistance index is calculated by dividing IC_50_ values on multidrug-resistant cell line M14/LCC6MDR1 by IC_50_ values on the matching sensitive parental cell line M14.

**Table 2:** *In vitro* growth inhibitory effects of C ring modified UC-112 analogs.

**ID**	**IC_50_ values** ± **SEM (μM)**				
	**A375**	**M14**	**M14/LCC6MDR1**	**PC-3**	**Resistance Index**
**6a**	2.7 ± 0.3	3.1± 0.3	15.3 ± 3.1	27.5 ± 2.0	4.9
**6b**	2.5 ± 0.5	1.9 ± 0.9	4.2 ± 0.2	3.4 ± 0.7	2.2
**6c**	8.4 ± 1.5	8.0 ± 2.2	> 30	> 30	>3.8
**6d**	1.0 ± 0.1	1.3 ± 0.6	ND^a^	ND	ND
**6e**	6.1 ± 0.7	5.1 ± 2.1	ND	ND	ND
**6f**	5.0 ± 0.3	5.9 ± 1.2	ND	ND	ND
**6g**	3.0 ± 0.5	4.9 ± 1.3	ND	ND	ND
**UC-112**	1.9 ± 0.6	2.1 ± 0.3	3.2 ± 0.5	1.6 ± 1.0	1.5

a ND: Not Determined.

**Table 3:** *In vitro* growth inhibitory effects of D ring modified UC-112 analogs.

**ID**	**IC_50_ values** ± **SEM (μM)**				
	**A375**	**M14**	**M14/LCC6MDR1**	**PC-3**	**Resistance Index**
**8a**	1.1 ± 0.1	1.3 ± 0.2	2.1 ± 0.6	1.3 ± 0.3	1.6
**8b**	5.0 ± 1.6	4.8 ± 0.5	3.5 ± 1.0	ND^a^	0.7
**UC-112**	1.9 ± 0.6	2.1 ± 0.3	3.2 ± 0.5	1.6 ± 1.0	1.5

a ND: Not Determined.

**Table 4:** *In vitro* growth inhibitory effects of UC-112 analogs with different chain lengths.

**ID**	**IC_50_ values** ± **SEM (μM)**			
	**A375**	**M14**	**M14/LCC6MDR1**	**Resistance Index**
**10a**	2.8 ± 0.9	2.9 ± 0.2	15.0 ± 2.5	5.2
**10b**	2.4 ± 0.7	2.3 ± 0.8	20.0 ± 1.6	8.7
**UC-112**	1.9 ± 0.6	2.1 ± 0.3	3.2 ± 0.5	1.5

### Effect of substitutions on the C ring of UC-112

The *in vitro* activity for compounds **4a-4l** which have substitutions on the C ring of UC-112 is shown in Table 1. Most compounds in this table are more active than the parent compound UC-112, suggesting that introduction of substitutions to the C ring moiety in UC-112 is favorable. Introducing a fluoro group (**4a**) to the phenyl ring caused the increase of activity in three of the four cancer cell lines tested(1.5 μM vs 1.9 μM in A375, 1.8 μM vs 2.1 μM in M14 and 0.7 μM vs 1.6 μM in PC-3). Introducing a chloro substitution (**4b**) resulted in even better activity in all four cell lines tested. A bromo substitution (**4c**) further increased activity. Introduction of an electron withdrawing group, trifluoromethyl (**4d**), also resulted in an increase in activity against all the cell lines. As the size of substitution increases from fluoro to bromo substitutions, a general trend of increased activity was observed (i.e. IC_50_ in M14 cell line. **4a**: 1.8 μM; **4b**: 1.1 μM; **4c**: 1.0 μM). This trend is also true for electron donating group substituted analogs **4e-4g** (i.e. IC_50_ in M14/LCC6MDR1 cell line. **4e**: 4.1 μM; **4f**: 2.7 μM; **4g**: 1.8 μM). But the *tert*-butyl group substituted compound **4h** does not further increase activity compared to *iso*-propyl group substituted compound **4g**, indicating there is probably a size limit for effective binding to survivin. In addition, several polar groups which are hydrogen bond acceptors were also tested. However, as Table 1 shows, compounds **4i- 4l** do not increase activity significantly compared to parent compound UC-112. Compounds **4j** and **4k** even had decreased activity compared to UC-112 (i.e. IC_50_ in M14/LCC6MDR1 cell line. **4j**: 12.1 μM; **4k**: 5.5 μM; **UC-112**: 3.2 μM), suggesting that a bulky substitution is unfavorable. Among all the compounds in this table, compounds **4c**, **4d** and **4g** are the most potent ones, indicating that the introduction of a hydrophobic group to the *para*-position of C ring is favorable for increased activity.

### Effect of replacing the phenyl ring of UC-112 with other groups

After we tried different substitutions on the phenyl ring of UC-112, we replaced the phenyl ring with other groups to further optimize UC-112. Here, we tried seven different groups which included furan ring, thiophene ring, pyridine ring, cyclohexyl ring, methyl group, ethyl group, and *n-*propyl group. The *in vitro* activity for the seven C ring modified UC-112 analogs is shown in Table 2. In this table, compounds **6a**, **6b** and **6c** were less active than UC-112 (i.e. IC_50_ in A375 cell line. **6a**: 2.7 μM; **6b**: 2.5 μM; **6c**: 8.4 μM; **UC-112**: 1.9 μM), indicating that replacing the phenyl ring with other heterocyclic rings decreases activity. Replacing the phenyl ring with a cyclohexyl ring resulted in an increase in activity in A375 and M14 cell lines. In order to see whether a ring is required at the C ring position of UC-112 for activity, compounds **6e**, **6f** and **6g** with only aliphatic chains were synthesized. All these compounds decreased activity compared to parent compound UC-112 (i.e. IC_50_ in A375 cell line. **6e**: 6.1 μM; **6f**: 5.0 μM; **6g**: 3.0 μM; **UC-112**: 1.9 μM), suggesting a ring system is required at the C ring position of UC-112. For compounds **6e**, **6f** and **6g,** there is a general trend for activity. As the length of the aliphatic chain increases, the activity increases as well. This trend is probably due to the existence of a hydrophobic pocket in the binding site, which is consistent with previous observation that introduction of a hydrophobic substitution with bigger space occupation on the phenyl ring is favorable for activity.

### Effect of D ring modification on UC-112

Following C ring modification in the above, we went on with D ring modification of UC-112. We tried to replace the pyrrolidine ring with two different cyclic amines: piperidine and morpholine at current stage. The *in vitro* activity for the two D ring modified compounds **8a** and **8b** is show in Table 3.Compound **8a** showed slightly better activity than the parent compound, while compound **8b** was less active than UC-112 (i.e. IC_50_ in A375 cell line. **8a**: 1.1 μM; **8b**: 5.0 μM; **UC-112**: 1.9 μM). This observation means the replacement of pyrrolidine ring in UC-112 with a piperidine ring maintains activity, while replacing pyrrolidine ring with morpholine ring decreases activity. Both compounds **8a** and **8b** can effectively inhibit the growth of resistant cell line M14/LCC6MDR1, suggesting that they can overcome the Pgp-mediated multiple drug resistance. The difference in activity between **8a** and **8b** indicates that the D ring position of UC-112 is an important site that further optimization can be carried out.

### Effect of chain length between oxygen and C ring of UC-112 analogs

The chain length between oxygen linker and C ring in the parent compound UC-112 is one carbon. We tried to test the importance of chain length by increasing the carbon numbers. Two compounds **10a** and **10b** were prepared and their *in vitro* activity is shown in Table 4. Both compounds **10a** and **10b** were less active than the parental compound, especially the activity against resistant cell line M14/LCC6MDR1 reduced from 3.2 to 15~20 μM, suggesting that increasing the chain length was not a favorable modification for increasing activity.

### Effect of linkers between C ring and D ring of UC-112 analogs

After the D ring modification, we went on to optimize the linker. We made two compounds **12a** and **12b** with two different linkers from the parent compound. The *in vitro* activity of those tow compounds is shown in Table 5. Compound **12a** which bears a sulfur linker shows increased activity compared with UC-112 (i.e. 1.4 μM vs 1.9 μM in A375 cell line), meaning sulfur linker here is favorable. Compound **12b** which contains a methylamine linker was less active (i.e. 7.1 μM vs 1.9 μM in A375 cell line), indicating that a methylamine linker is unfavorable. Both compounds **12a** and **12b** have small resistance indexes. Their activity in the resistant cell line M14/LCC6MDR1 is comparable with that in the parental cell line M14. While two compounds can’t determine the optimized linker, at least they give us some information about the activity requirement of the linker which is very useful for further optimization. Further optimization of the linker is now currently being examined.

**Table 5:** *In vitro* growth inhibitory effects of UC-112 analogs with different linkers.

**ID**	**IC_50_ values ± SEM (μM)**				
	**A375**	**M14**	**M14/LCC6MDR1**	**PC-3**	**Resistance Index**
**12a**	1.4 ± 0.3	1.4 ± 0.2	2.9 ± 0.6	1.1 ± 0.1	2.1
**12b**	7.1 ± 0.7	6.2 ± 1.4	8.0 ± 1.0	2.5 ± 0.6	1.3
**UC-112**	1.9 ± 0.6	2.1 ± 0.3	3.2 ± 0.5	1.6 ± 1.0	1.5

### UC-112 analogs can overcome multidrug resistance

In order to determine whether the new analogs we made can overcome multidrug resistance (MDR), we compared the activity of those analogs against multidrug-resistant melanoma cells (M14/LCCMDR1) and their parental sensitive cancer cells (M14). This pair of cell lines have been well validated and widely used to assess abilities of drugs overcoming Pgp-mediated MDR.[23–25] Our three best compounds **4c**, **4d** and **4g** together with the most successful small molecule survivin inhibitor YM155 were tested on both the MDR melanoma cells and their parental cells (Table 1). The resistance index is calculated by dividing the IC_50_ value of the resistant cell line by IC_50_ value of the sensitive parental cell line. So the smaller this value, the better resistance overcoming effect obtained. As this table shows, compounds **4c**, **4d** and **4g** all have very small resistance indexes (1.8, 1.9 and 2.3 respectively). Their activity in the resistant cell line is comparable with that in the parental cell line. For existing survivin inhibitor, YM155, although it is very potent in parental cell line (IC_50_ at 3 nM), its activity in resistant cell line is considerably lower (IC_50_ is higher than 10 μM, resistance index is higher than 2941). This data indicate that the new UC-112 analogs can circumvent Pgp-mediated multi-drug resistance and are distinct from that of YM-155.

### UC-112 analogs show good anti-proliferation effects with selectivity in NCI-60 cell line screening

**Fig 7:**
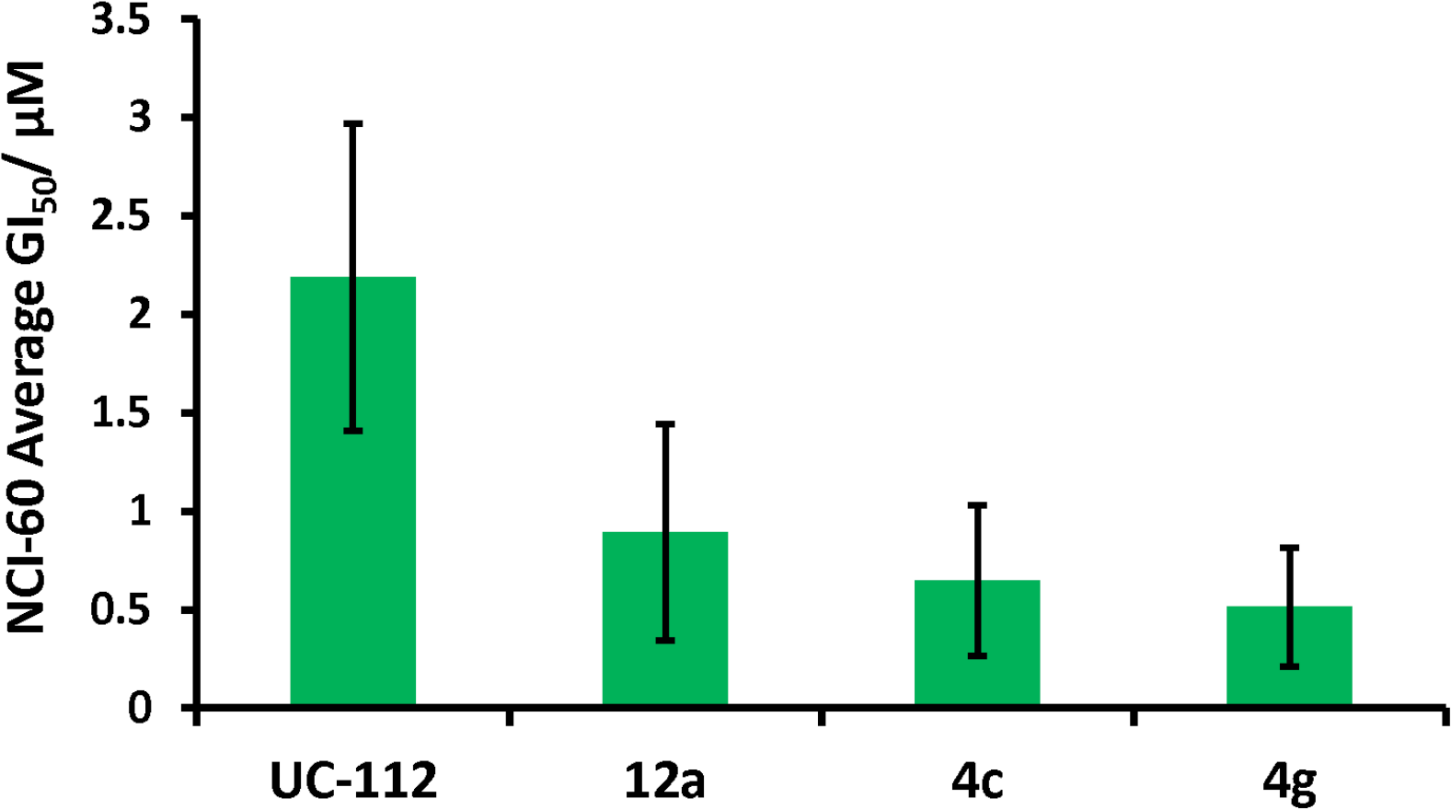
Average GI_50_ data for UC-112, compound 12c, 4c and 4g tested in NCI-60 anti-proliferative screening. Data is shown with mean ± SD as bar graph.

UC-112 and three potent analogs **4c**, **4g** and **12a** were submitted to NCI for its one-concentration (10 μM) screening against the NCI-60 cell lines. All four compounds showed good activity and were selected for the subsequent five doses testing to determine their growth inhibition potency (GI_50_) in the NCI-60 cell lines. As shown in Fig 7, structure modification from UC-112 to our best compound **4g** has improved the average GI_50_ by nearly four times (2.2 μM for UC-112 *vs*. 0.5 μM for **4g**).

**Fig 8:**
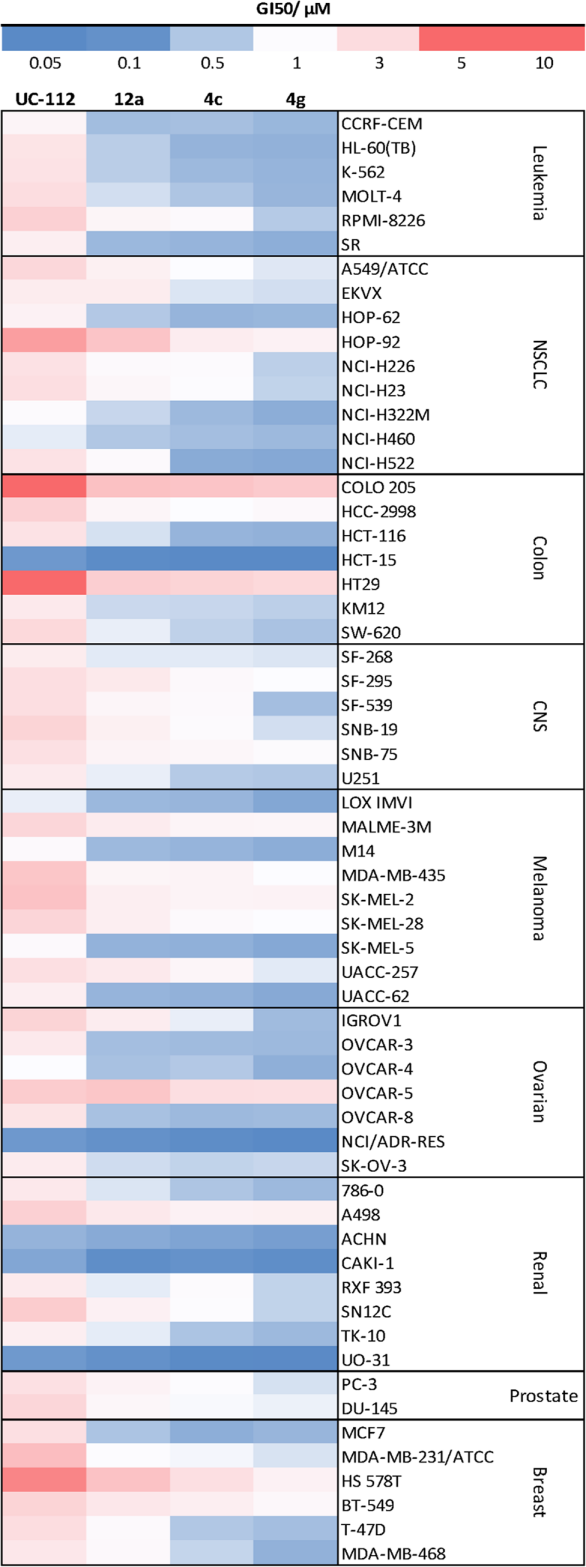
Heat map showing the GI_50_ values (nM) for UC-112 and three analogs in the NCI-60 screening. High intensity (blue) cells indicate high activity and low intensity (red) cells indicate low activity. Average GI_50_ values were calculated for each compound, separately.

The heat map in Fig 8 summarizes the compound growth inhibition pattern which is characterized by the GI_50_ mean values from NCI-60 screening (S1 Fig, S2 Fig, S3 Fig, and S4 Fig). UC-112 and its new analogs showed interesting selective growth inhibition behavior within the NCI 60 cell lines. The GI_50 _value of compound **4g** in renal cancer cell line UO-31 was as low as 52.5 nM. Interestingly, two other cell lines, HCT-15 (colon cancer) and NCI/ADR-RES (ovarian cancer), were also particularly sensitive to the treatment of UC-112 and its analogs, with lowest GI_50_ value (highest activity) for compound **4g** at 46.8 nM and 50.1 nM, respectively. Since colorectal adenocarcinoma HCT-15 cells intrinsically expresses moderate levels of Pgp, multidrug-resistance-associated protein (MRP) and lung-resistance-associated protein (LRP) [26], and ovarian cancer NCI/ADR-RES cells are naturally over-expressing MDR1 and resistant to various chemotherapies including doxorubicin [27], these data have supported our findings that UC-112 and its analogs could effectively overcome the multidrug resistance *in vitro*.

### Compound 4g possesses good drug-like properties

Adsorption, distribution, metabolism and elimination (ADME) properties are key elements in small molecule drug development. Poor ADME property is one of the major contributors of failure in advancing new compounds towards drug approval. To preliminarily evaluate the overall drug-like properties for the UC-112 scaffold, we sent our best compound **4g** to an external contract research company (Eurofins Panlabs Inc., Redmond, WA) to determine its aqueous solubility and *in vitro* ADME properties. The aqueous solubility for compound **4g** was tested in PBS solution at pH 7.4 (Table 6) and the result showed that compound **4g** had very good aqueous solubility of 148.7 μM. The *in vitro* microsomal stability for compound **4g** was determined in human liver microsomes. The result showed that it had a reasonable half life time at 51 minutes with a medium clearance rate at 136.6 μL/min/mg. Drug metabolism studies using cytochrome P450 inhibition by compound **4g** (Table 7) showed weak inhibition for most cytochrome P450 enzymes (less than 30%) except CYP2D6. This study suggests that the UC-112 scaffold demonstrated by compound **4g** has good drug-like properties, which clearly warrants its further development.

**Table 6:** Aqueous solubility and *in vitro* metabolism properties of compound 4g.

**ID**	**Solubility** ^a^ **(μM)**	**Half-Life** ^b^ **(min)**	**Clint (μL/min/mg)**
**4g**	148.7	51.0	136.6

a test concentration is 200 μM

b test concentration is 100 nM.

**Table 7:** Cytochrome P450 inhibition effects of compound 4g.

**CYP enzyme**	**Substrate**	**% inhibition of control values**
CYP1A	phenacetin	18
CYP2B6	bupropion	21
CYP2C8	paclitaxel	21
CYP2C9	diclofenac	17
CYP2C19	omeprazole	45
CYP2D6	dextromethorphan	87
CYP3A	midazolam	3
CYP3A	testosterone	29

### Mechanism of action studies

**Fig 9:**
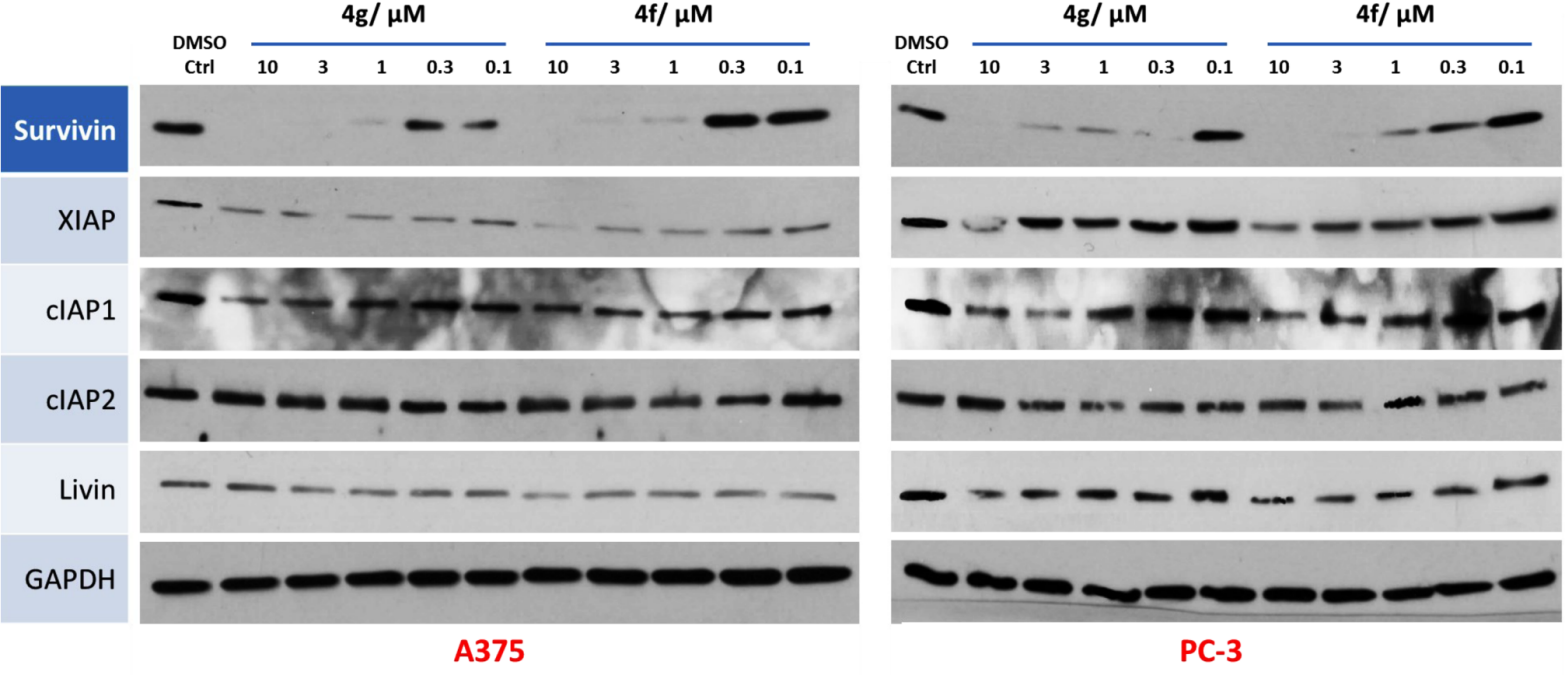
Western blotting assay of A375 and PC-3 cells treated with gradient increasing dose of 4f or 4g for 24 h. Left panel is in A375 cancer cell line. Right panel is in PC-3 cancer cell line.

**Fig 10:**
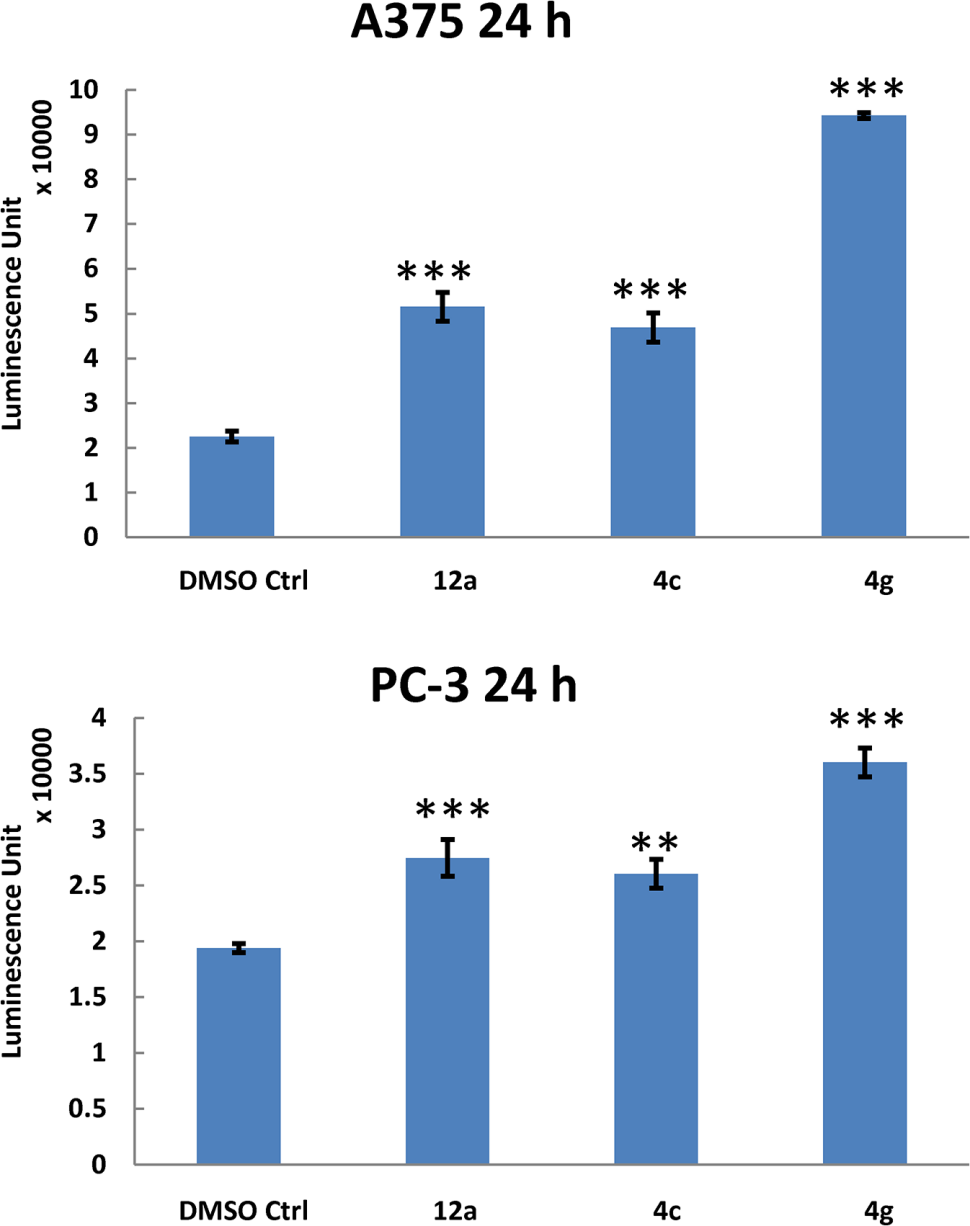
Relative *in vitro* caspases 3/7 activity of human melanoma A375 or human prostate cancer PC-3 cells were evaluated after treatment of UC-112 analogs (1 μM) for 24 h (*N* = 3). The luminescence unit data was adjusted according to the cell viability results read from the same well in 96-well plate. ***P < 0.001, **P < 0.01 compared with corresponded results from control group.

In our recently report, [19] UC-112 induces apoptosis by selectively inhibiting the expression of survivin in cancer cells. In order to determine whether the new UC-112 analogs maintained the same mechanism of action, we performed the caspases activation and western blotting assay for two potent new analogs **4f** and **4g** in two cancer cell lines (Fig 9). Both compounds **4f** and **4g** dose-dependently suppressed survivin level in these two cancer cell lines, while the levels of other IAPs were minimally affected. The presence of compound **4g** at 300 nM reduced survivin levels over 50% in both A375 and PC-3 cells as shown in the lane density data quantified by ImageJ software (S5 Fig). The IC_50_ values for survivin inhibition were estimated to be in the low nanomolar range. Furthermore, incubation of compound **12c**, **4c** or **4g** at concentration of 1 μM for 24 h significantly activated the executioner caspase 3/7 up to 4 folds higher than the DMSO control group (Fig 10). This is consistent with the expected enhanced apoptosis induction capability due to survivin inhibition.

### Molecular modeling study

**Fig 11:**
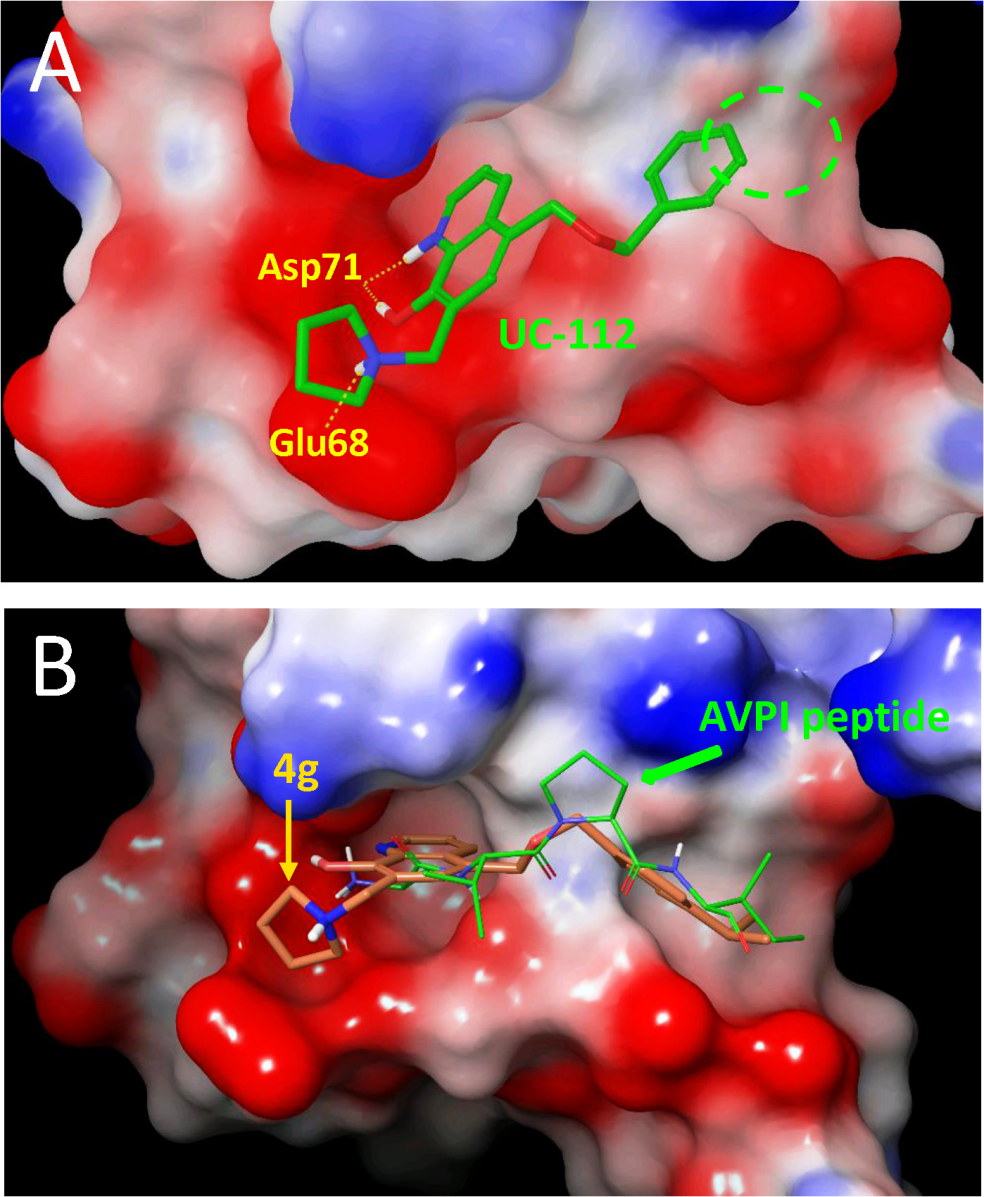
Potential binding pose of UC-112 and compound 4g in the SMAC N-terminus tetra-peptide AVPI binding site of survivin crystal structure (PDB entry: 3UIH). The surface of AVPI binding site in survivin was colored according to residue charge (blue for positive while red for negative). (a) UC-112 (green tube) formed hydrogen bonds with residue Asp71 and Glu68 on the survivin protein BIR domain. (b) Compound **4g** (orange tube) displayed similar binding pose with UC-112 but had better occupied the grove toward the N-terminus of survivin protein. And this pose was overlapping well with the binding mode of native ligand, SMAC AVPI (green stick).

To understand the observed improvement in potency of compound **4g** over its parental compound UC-112, we developed a molecular model and performed the molecular docking study using the complex of human survivin-SMAC AVPI (PDB entry: 3UIH). As shown in Fig 11, UC-112 displayed several interesting interactions with the survivin protein BIR domain: (1) two hydrogen bonding interactions between the A/B-ring of UC-112 and residue Asp71; (2) one hydrogen bonding interaction between the D-ring nitrogen and residue Glu68; and (3) an π- π stacking interaction between the A/B-ring of UC-112 and residue Typ67 (Fig 11A). Examination of this proposed binding pose clearly suggests that the un-substituted phenyl ring of UC-112 failed to occupy a hydrophobic groove (cycled with green dash line in Fig 11A), and a properly sized, non-polar *para*-substitution (e.g., an isopropyl moiety as in compound **4g**) to this ring would fill this groove and also provide excellent overlap with the bioactive AVPI peptide (Fig 11B). This is consistent with our experimental observations. Further refinement to this model, and ultimately an X-ray crystal structure will greatly facilitate the future optimization of this scaffold.

### Drug affinity responsive target stability (DARTS) assay

**Fig 12:**
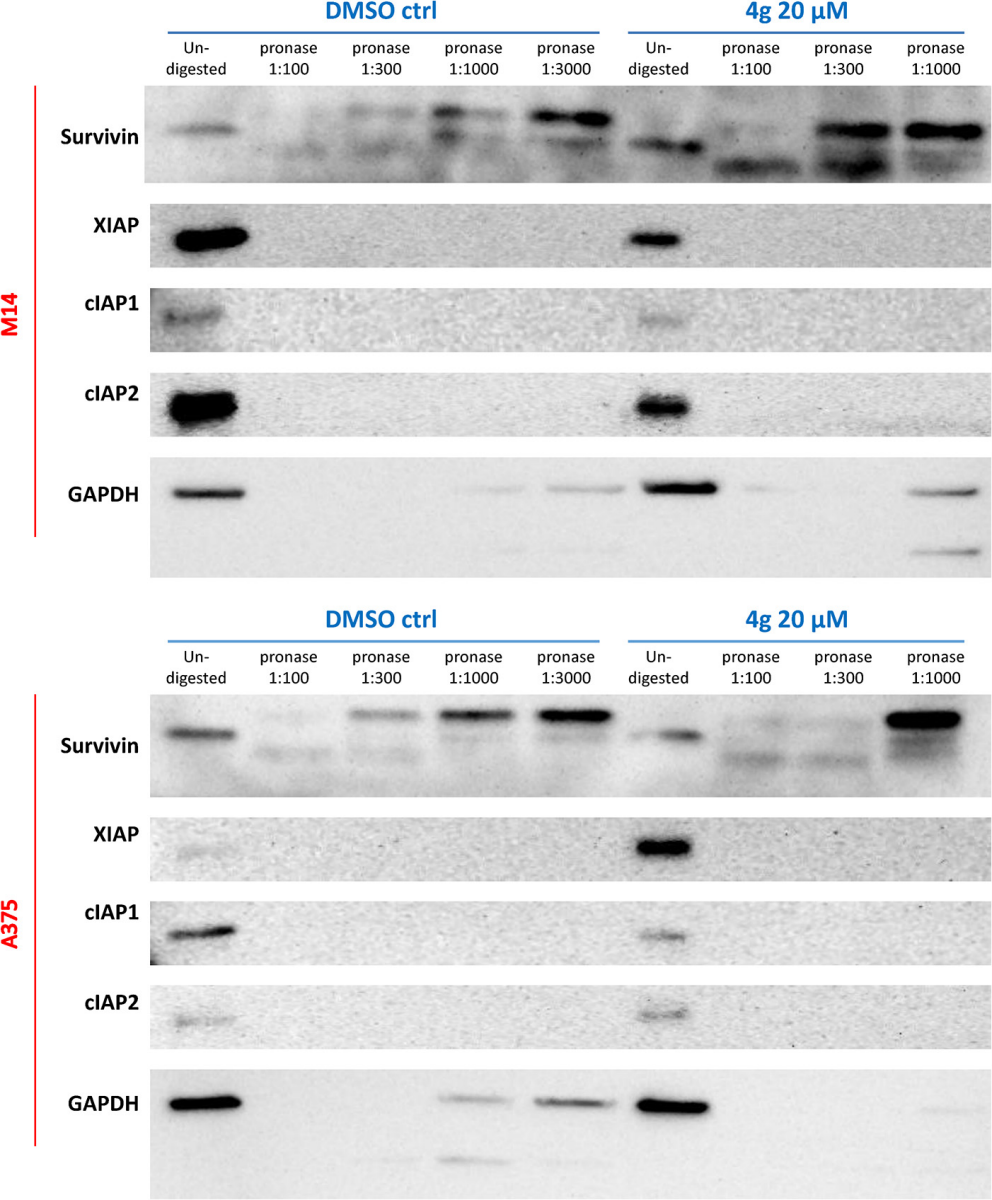
Representative drug affinity responsive target stability (DARTS) results for pronase-digested A375 or M14 cell lysates. Immunoblotting showed protection of the target protein, survivin, by incubation with compound **4g** at the concentration of 20 μM, whereas digestion of the non-target proteins like GAPDH was unchanged.

To investigate that whether the survivin down-regulation effect is caused by direct interaction between survivin protein and UC-112 analogs, we performed DARTS assay which is a well-established target identification method [28–31]. DARTS assay relies on the increasing of proteolysis resistance of the target protein generated by the interaction with small molecular ligand. We utilized immunoblotting to detect the abundances of several proteins including survivin in M14 or A375 cell lysates either undigested or digested by different concentrations of pronase. Representative data shown in Fig 12 clearly indicated that the protease susceptibility of survivin is significantly reduced in cell lysates pre-treated with 20 μM **4g**, comparing with the GAPDH control group. Consistent with its selective survivin inhibition, **4g** has negligible effects to protease susceptibility for other IAPs such as XIAP, cIAP1 or cIAP2. This observation suggested a possible direct interaction between **4g** and survivin protein in cell lysates that will be confirmed by X-ray crystallography in the future.

### *In vivo* anti-tumor efficacy assessment

**Fig 13:**
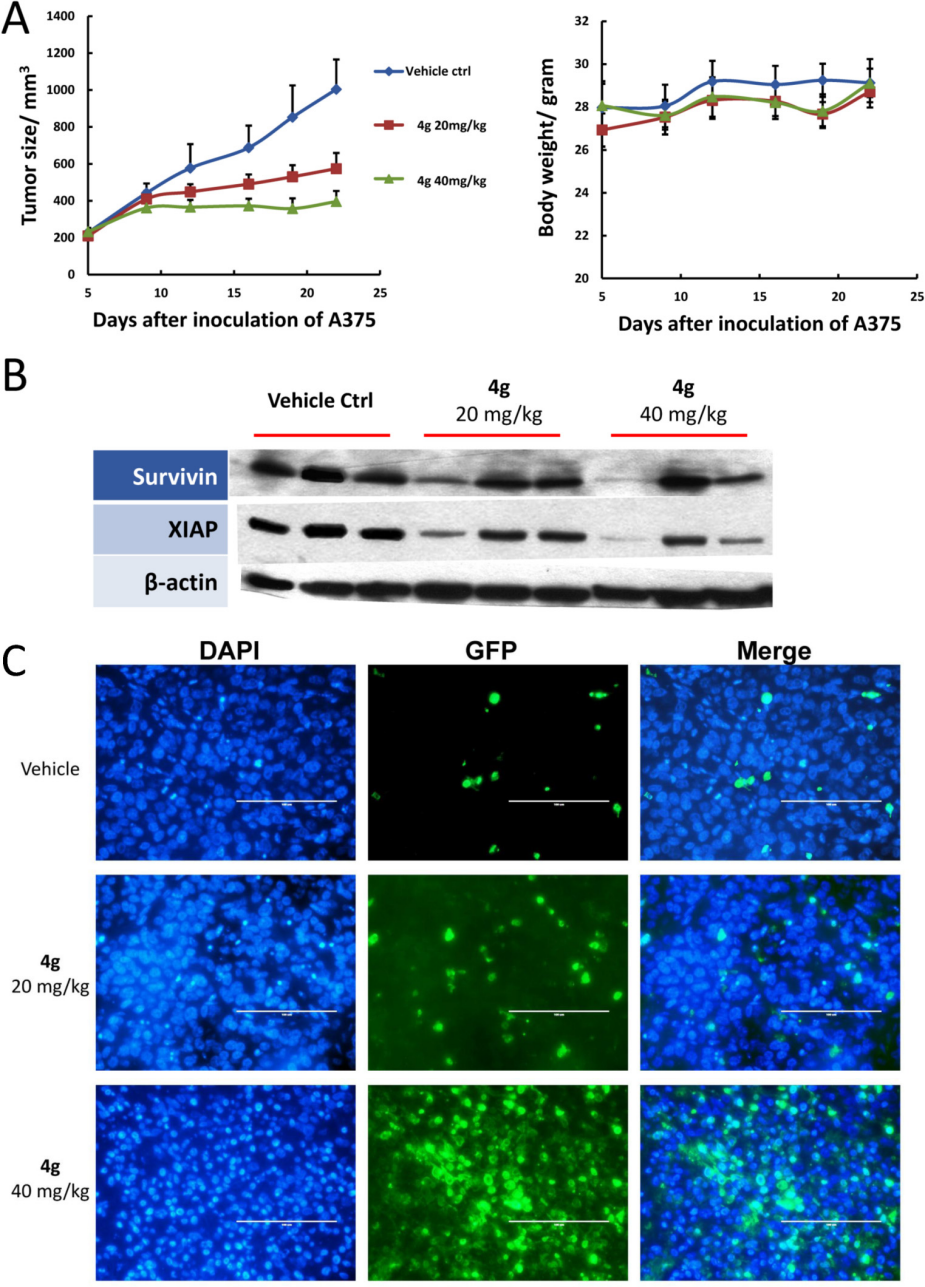
*In vivo* anti-tumor efficacy of compound 4g (*N* = 6). (a) **4g** effectively inhibited the growth of A375 xenograft tumor after three weeks continuous treatment (i.p. injection) in a dose-dependent manner (left panel) without causing obvious decrease of mice body weight (right panel). (b) Western blotting results on the A375 xenograft tumor tissues (each lane represents one single mice). (c) Representative images of TUNEL assay using the formalin-fixed tumor sections.

Since our *in vitro* study showed that compound **4g** has the highest anti-proliferative potency in this series of UC-112 analogs, we selected **4g** to test its *in vivo* efficacy against tumor growth in a human melanoma A375 xenograft model through *i*.*p* injection. As shown in Fig 13A, compound **4g** inhibited the growth of A375 xenograft tumor in a dose-dependent manner during the three weeks of continuous treatment. The tumor growth in compound **4g** 20 mg/kg and 40 mg/kg treatment group is 53% and 79% slower than the vehicle control group, respectively. Furthermore, Western blotting analysis revealed that the expression levels of survivin and XIAP protein decreased in tumor tissues which were freshly collected from compound **4g** treated groups (Fig 13B). Terminal deoxynucleotidyl transferase dUTP nick end labeling (TUNEL) assay, which measured the nuclear DNA fragmentation, showed that a high dose (40 mg/kg) of compound **4g** treatment caused the widely-spread cell apoptosis inside the tumor tissues (Fig 13C).

## Materials and Methods

### General

All animal studies described in this manuscript have been approved by the IACUC at the University of Tennessee Health Science Center. All reagents were purchased from Sigma-Aldrich Chemical Co., Alfa Aesar (Ward Hill, MA), and AK Scientific (Mountain View, CA) and were used without further purification. Routine thin layer chromatography (TLC) was performed on aluminum-backed Uniplates (Analtech, Newark, DE). NMR spectra were obtained on a Varian Inova-500 spectrometer (Agilent Technologies, Santa Clara, CA) or a Bruker Ascend 400 (Billerica, MA) spectrometer. Chemical shifts are reported as parts per million (ppm) relative to TMS in CDCl_3_. High Resolution Mass spectra were collected in positive detection mode on a Waters Xevo G2-S Tof instrument equipped with an electron-spray ionization (ESI) source (Milford, MA).

### Synthesis

#### Preparation of 5-chloromethyl-8-quinolinol hydrochloride (2)

A mixture of 5.84 g (40.0 mmol) of 8-quinolinol, 50 ml of concentrated hydrochloric acid, and 6.4 ml of 37% formaldehyde was treated with 0.6 g of zinc chloride and stirred for 12 h. The mixture was filtered, washed with copious acetone and dried to give compound **2** as a yellow solid (7.2g, 78%). ^1^H NMR (400 MHz, Deuterium Oxide) δ 9.12 (dd, *J* = 8.7, 1.4 Hz, 1H), 8.88 (dd, *J* = 5.5, 1.4 Hz, 1H), 7.97 (dd, *J* = 8.7, 5.4 Hz, 1H), 7.59 (d, *J* = 8.0 Hz, 1H), 7.24 (d, *J* = 7.9 Hz, 1H), 4.93 (s, 2H).

#### General procedure for the synthesis of compounds (3a-3l)

Method A(only for **3a**,**3f** and **3g**): 5-chloromethyl-8-quinolinol hydrochloride **2** (1.15 g, 5 mmol) was suspended in substituted benzyl alcohol **3** (25 mmol) and the mixture was heated at 90°C for 2 h, during which time the solution became completely homogeneous. The solution was then poured to 200 ml diethyl ether. The corresponding hydrochloride salt was recovered by filtration as a bright yellow solid. The yellow solid was then suspended in diethyl ether and aqueous NH_4_OH (2.5% v/v, 7.5 mL, ~1.2 equiv.) was added drop by drop under vigorous stirring until the aqueous phase was basic (pH 8–10). The organic phase was washed with water, dried with anhydrous sodium sulfate and evaporated to dryness to afford the desired ethers.

Method B (for **3b-e** and **3h-l**): To a solution of substituted benzyl alcohol **3**(6 mmol) in anhydrous THF(30ml) was added sodium hydride (60% dispersion in mineral oil, 0.72g, 18 mmol) at 0 oC. The suspension was stirred at 0 oC for 30 minutes. Salt **2** (1.15g, 5mmol) was added to the suspension. The mixture was stirred at r.t for 3 hours. Water was added to the suspension, the mixture became homogeneous. The mixture was extracted by ethyl acetate and washed with brine, dried over anhydrous sodium sulfate and concentrated to get the crude. The crude compound was purified by flash chromatography (ethyl acetate: hexane 3:1).

#### 5-(((4-fluorobenzyl)oxy)methyl)quinolin-8-ol(3a)

^1^H NMR (400 MHz, Chloroform-*d*) δ 8.81 (dd, *J* = 4.2, 1.6 Hz, 1H), 8.47 (dd, *J* = 8.5, 1.5 Hz, 1H), 7.49 (dd, *J* = 8.5, 4.2 Hz, 1H), 7.41 (d, *J* = 7.8 Hz, 1H), 7.32 – 7.27 (m, 2H), 7.11 (d, *J* = 7.7 Hz, 1H), 7.06 – 6.98 (m, 2H), 4.87 (s, 2H), 4.50 (s, 2H).

#### 5-(((4-chlorobenzyl)oxy)methyl)quinolin-8-ol(3b)

^1^H NMR (400 MHz, Chloroform-*d*) δ 8.83 (dd, *J* = 4.2, 1.6 Hz, 1H), 8.51 (dd, *J* = 8.5, 1.6 Hz, 1H), 7.52 (dd, *J* = 8.5, 4.2 Hz, 1H), 7.44 (d, *J* = 7.7 Hz, 1H), 7.36 – 7.31 (m, 5H), 7.14 (d, *J* = 7.7 Hz, 1H), 4.90 (s, 2H), 4.53 (s, 2H).

#### 5-(((4-bromobenzyl)oxy)methyl)quinolin-8-ol(3c)

^1^H NMR (400 MHz, Chloroform-*d*) δ 8.81 (dd, *J* = 4.2, 1.6 Hz, 1H), 8.47 (dd, *J* = 8.5, 1.6 Hz, 1H), 7.51 – 7.45 (m, 3H), 7.41 (d, *J* = 7.7 Hz, 1H), 7.22 – 7.17 (m, 2H), 7.11 (d, *J* = 7.7 Hz, 1H), 4.87 (s, 2H), 4.48 (s, 2H).

#### 5-(((4-(trifluoromethyl)benzyl)oxy)methyl)quinolin-8-ol(3d)

^1^H NMR (400 MHz, Chloroform-*d*) δ 8.75 (dd, *J* = 4.2, 1.6 Hz, 1H), 8.45 (dd, *J* = 8.5, 1.6 Hz, 1H), 7.56 – 7.49 (m, 2H), 7.45 (dd, *J* = 8.5, 4.3 Hz, 1H), 7.36 (ddd, *J* = 7.8, 2.0, 1.2 Hz, 3H), 7.08 (d, *J* = 7.7 Hz, 1H), 4.85 (s, 2H), 4.51 (s, 2H).

#### 5-(((4-methylbenzyl)oxy)methyl)quinolin-8-ol(3e)

^1^H NMR (500 MHz, Chloroform-*d*) δ 8.72 (dd, *J* = 4.2, 1.6 Hz, 1H), 8.40 (dd, *J* = 8.5, 1.6 Hz, 1H), 7.40 (dd, *J* = 8.5, 4.1 Hz, 1H), 7.34 (d, *J* = 7.7 Hz, 1H), 7.15 (d, *J* = 7.9 Hz, 2H), 7.09 (d, *J* = 7.8 Hz, 2H), 7.03 (d, *J* = 7.7 Hz, 1H), 4.78 (s, 2H), 4.44 (s, 2H), 2.28 (s, 3H).

#### 5-(((4-ethylbenzyl)oxy)methyl)quinolin-8-ol(3f)

^1^H NMR (400 MHz, Chloroform-*d*) δ 8.72 (dd, *J* = 4.2, 1.6 Hz, 1H), 8.41 (dd, *J* = 8.5, 1.5 Hz, 1H), 7.40 (dd, *J* = 8.5, 4.2 Hz, 1H), 7.35 (d, *J* = 7.7 Hz, 1H), 7.17 (d, *J* = 2.1 Hz, 2H), 7.11 (d, *J* = 8.0 Hz, 2H), 7.03 (d, *J* = 7.7 Hz, 1H), 4.79 (s, 2H), 4.44 (s, 2H), 2.58 (q, *J* = 7.6 Hz, 2H), 1.16 (t, *J* = 7.6 Hz, 3H).

#### 5-(((4-isopropylbenzyl)oxy)methyl)quinolin-8-ol(3g)

^1^H NMR (400 MHz, Chloroform-*d*) δ 8.72 (dd, *J* = 4.2, 1.6 Hz, 1H), 8.41 (dd, *J* = 8.5, 1.6 Hz, 1H), 7.40 (dd, *J* = 8.5, 4.2 Hz, 1H), 7.35 (d, *J* = 7.7 Hz, 1H), 7.18 (d, *J* = 4.2 Hz, 2H), 7.14 (d, *J* = 8.2 Hz, 2H), 7.03 (d, *J* = 7.7 Hz, 1H), 4.80 (s, 2H), 4.44 (s, 2H), 2.83 (p, *J* = 6.9 Hz, 1H), 1.17 (d, *J* = 7.0 Hz, 6H).

#### 5-(((4-(tert-butyl)benzyl)oxy)methyl)quinolin-8-ol(3h)

^1^H NMR (400 MHz, Chloroform-*d*) δ 8.67 (dd, *J* = 4.2, 1.6 Hz, 1H), 8.37 (dd, *J* = 8.5, 1.6 Hz, 1H), 7.35 (dd, *J* = 8.5, 4.2 Hz, 1H), 7.31 (d, *J* = 7.7 Hz, 1H), 7.25 (d, *J* = 8.5 Hz, 2H), 7.17–7.13 (m, 2H), 6.98 (d, *J* = 7.7 Hz, 1H), 4.76 (s, 2H), 4.40 (s, 2H), 1.20 (s, 9H).

#### 5-(((4-methoxybenzyl)oxy)methyl)quinolin-8-ol(3i)

^1^H NMR (400 MHz, Chloroform-*d*) δ 8.80 (dd, *J* = 4.2, 1.6 Hz, 1H), 8.47 (dd, *J* = 8.5, 1.6 Hz, 1H), 7.48 (dd, *J* = 8.5, 4.2 Hz, 1H), 7.41 (d, *J* = 7.8 Hz, 1H), 7.28–7.23 (m, 2H), 7.10 (d, *J* = 7.7 Hz, 1H), 6.93–6.82 (m, 2H), 4.84 (s, 2H), 4.48 (s, 2H), 3.81 (s, 3H).

#### 5-(((4-(benzyloxy)benzyl)oxy)methyl)quinolin-8-ol(3j)

^1^H NMR (500 MHz, Chloroform-*d*) δ 8.84 (d, *J* = 4.2 Hz, 1H), 8.51 (d, *J* = 8.5 Hz, 1H), 7.53–7.41 (m, 6H), 7.37 (t, *J* = 7.3 Hz, 1H), 7.34–7.27 (m, 2H), 7.16 (d, *J* = 7.6 Hz, 1H), 7.06–6.98 (m, 2H), 5.12 (s, 2H), 4.89 (s, 2H), 4.53 (s, 2H).

#### N-(4-(((8-hydroxyquinolin-5-yl)methoxy)methyl)phenyl)acetamide(3k)

^1^H NMR (400 MHz, DMSO-*d*6) δ 9.93 (s, 1H), 9.86 (s, 1H), 8.87 (dd, *J* = 4.1, 1.6 Hz, 1H), 8.47 (dd, *J* = 8.6, 1.6 Hz, 1H), 7.60 (dd, *J* = 8.6, 4.2 Hz, 1H), 7.57–7.52 (m, 2H), 7.44 (d, *J* = 7.8 Hz, 1H), 7.28–7.21 (m, 2H), 7.02 (d, *J* = 7.7 Hz, 1H), 4.83 (s, 2H), 4.47 (s, 2H), 2.03 (s, 3H).

#### 5-(((3,4,5-trimethoxybenzyl)oxy)methyl)quinolin-8-ol (3l)

^1^H NMR (400 MHz, Chloroform-*d*) δ 8.85 (dd, *J* = 4.3, 1.5 Hz, 1H), 8.57 (dd, *J* = 8.5, 1.5 Hz, 1H), 7.53 (dd, *J* = 8.5, 4.2 Hz, 1H), 7.47 (d, *J* = 7.8 Hz, 1H), 7.19 (d, *J* = 7.6 Hz, 1H), 6.56 (s, 2H), 4.92 (s, 2H), 4.50 (s, 2H), 3.86 (s, 3H), 3.84 (s, 6H)

#### General Procure for the preparation of compounds (4a-4l)

An equimolar mixture of the substrates **3**, paraformaldehyde, and the pyrrolidine in anhydrous ethanol (30 mL) was refluxed for 4 hours under argon. After cooling, the solvent was evaporated under reduced pressure. The crude compound was purified by flash chromatography (Dichloromethane: methanol 20: 1).

#### 5-(((4-fluorobenzyl)oxy)methyl)-7-(pyrrolidin-1-ylmethyl)quinolin-8-ol(4a)

^1^H NMR (400 MHz, Chloroform-*d*) δ 8.91 (dd, *J* = 4.1, 1.6 Hz, 1H), 8.40 (dd, *J* = 8.5, 1.7 Hz, 1H), 7.44 (dd, *J* = 8.5, 4.2 Hz, 1H), 7.35 – 7.30 (m, 2H), 7.26 (s, 1H), 7.10 – 6.97 (m, 2H), 4.87 (s, 2H), 4.55 (s, 2H), 4.02 (s, 2H), 2.84 – 2.61 (m, 4H), 2.01 – 1.79 (m, 4H). HRMS (ESI): m/z calculated for C_22_H_23_FN_2_O_2_ + H^+^ [M + H^+^]: 367.1822; Found: 367.1828.

#### 5-(((4-chlorobenzyl)oxy)methyl)-7-(pyrrolidin-1-ylmethyl)quinolin-8-ol(4b)

^1^H NMR (400 MHz, Chloroform-*d*) δ 8.82 (dd, *J* = 4.1, 1.6 Hz, 1H), 8.30 (dd, *J* = 8.5, 1.7 Hz, 1H), 7.34 (dd, *J* = 8.5, 4.1 Hz, 1H), 7.27 – 7.22 (m, 2H), 7.21 – 7.17 (m, 2H), 7.15 (s, 1H), 4.77 (s, 2H), 4.45 (s, 2H), 3.92 (s, 2H), 2.70 – 2.56 (m, 4H), 1.88 – 1.74 (m, 4H). HRMS (ESI): m/z calculated for C_22_H_23_ClN_2_O_2_ + H^+^ [M + H^+^]: 383.1526; Found: 383.1521.

#### 5-(((4-bromobenzyl)oxy)methyl)-7-(pyrrolidin-1-ylmethyl)quinolin-8-ol(4c)

^1^H NMR (400 MHz, Chloroform-*d*) δ 8.89 (dd, *J* = 4.2, 1.7 Hz, 1H), 8.38 (dd, *J* = 8.5, 1.7 Hz, 1H), 7.49 – 7.44 (m, 2H), 7.42 (dd, *J* = 8.5, 4.1 Hz, 1H), 7.25 (s, 1H), 7.23 – 7.17 (m, 2H), 4.85 (s, 2H), 4.51 (s, 2H), 4.01 (s, 2H), 2.84 – 2.64 (m, 4H), 1.97 – 1.81 (m, 4H). HRMS (ESI): m/z calculated for C_22_H_23_BrN_2_O_2_ + H^+^ [M + H^+^]: 427.1021; Found: 427.1015.

#### 7-(pyrrolidin-1-ylmethyl)-5-(((4-(trifluoromethyl)benzyl)oxy)methyl)quinolin-8-ol(4d)

^1^H NMR (500 MHz, Chloroform-*d*) δ 8.91 (dd, *J* = 4.1, 1.6 Hz, 1H), 8.42 (dd, *J* = 8.5, 1.7 Hz, 1H), 7.61 (d, *J* = 8.0 Hz, 2H), 7.49 – 7.40 (m, 3H), 7.27 (d, *J* = 3.1 Hz, 1H), 4.90 (s, 2H), 4.62 (s, 2H), 4.02 (s, 2H), 2.82 – 2.68 (m, 4H), 1.97 – 1.85 (m, 4H). HRMS (ESI): m/z calculated for C_23_H_23_F_3_N_2_O_2_ + H^+^ [M + H^+^]: 417.1790; Found: 417.1792.

#### 5-(((4-methylbenzyl)oxy)methyl)-7-(pyrrolidin-1-ylmethyl)quinolin-8-ol(4e)

^1^H NMR (500 MHz, Chloroform-*d*) δ 8.80 (dd, *J* = 4.2, 1.7 Hz, 1H), 8.31 (dd, *J* = 8.5, 1.7 Hz, 1H), 7.34 (dd, *J* = 8.5, 4.1 Hz, 1H), 7.23 (s, 1H), 7.16 (d, *J* = 7.9 Hz, 2H), 7.09 (d, *J* = 7.7 Hz, 2H), 4.75 (s, 2H), 4.46 (s, 2H), 3.96 (s, 2H), 2.80 – 2.65 (m, 4H), 2.28 (s, 3H), 1.88–1.78 (m, 4H). HRMS (ESI): m/z calculated for C_23_H_26_N_2_O_2_ + H^+^ [M + H^+^]: 363.2073; Found: 363.2078.

#### 5-(((4-ethylbenzyl)oxy)methyl)-7-(pyrrolidin-1-ylmethyl)quinolin-8-ol(4f)

^1^H NMR (400 MHz, Chloroform-*d*) δ 8.81 (dd, *J* = 4.2, 1.7 Hz, 1H), 8.30 (dd, *J* = 8.5, 1.7 Hz, 1H), 7.33 (dd, *J* = 8.5, 4.1 Hz, 1H), 7.20 (s, 1H), 7.18 (d, *J* = 5.4 Hz, 2H), 7.12 (d, *J* = 8.1 Hz, 2H), 4.76 (s, 2H), 4.47 (s, 2H), 3.93 (s, 2H), 2.70 – 2.62 (m, 4H), 2.58 (q, *J* = 7.6 Hz, 2H), 1.88 – 1.74 m, 4H), 1.16 (t, *J* = 7.6 Hz, 3H). HRMS (ESI): m/z calculated for C_24_H_28_N_2_O_2_ + H^+^ [M + H^+^]: 377.2229; Found: 377.2240.

#### 5-(((4-isopropylbenzyl)oxy)methyl)-7-(pyrrolidin-1-ylmethyl)quinolin-8-ol(4g)

^1^H NMR (400 MHz, Chloroform-*d*) δ 8.89 (dd, *J* = 4.1, 1.6 Hz, 1H), 8.38 (dd, *J* = 8.5, 1.6 Hz, 1H), 7.40 (dd, *J* = 8.5, 4.1 Hz, 1H), 7.29 (s, 1H), 7.26 – 7.20 (m, 4H), 4.85 (s, 2H), 4.55 (s, 2H), 4.00 (s, 2H), 2.92 (p, *J* = 6.9 Hz, 1H), 2.78 – 2.60 (m, 4H), 1.96 – 1.80 (m, 4H), 1.25 (d, *J* = 6.9 Hz, 6H). HRMS (ESI): m/z calculated for C_25_H_30_N_2_O_2_ + H^+^ [M + H^+^]: 391.2386; Found: 391.2377.

#### 5-(((4-(tert-butyl)benzyl)oxy)methyl)-7-(pyrrolidin-1-ylmethyl)quinolin-8-ol(4h)

^1^H NMR (400 MHz, Chloroform-*d*) δ 8.81 (dd, *J* = 4.1, 1.6 Hz, 1H), 8.31 (dd, *J* = 8.5, 1.7 Hz, 1H), 7.36 – 7.28 (m, 3H), 7.23 – 7.19 (m, 2H), 4.78 (s, 2H), 4.47 (s, 2H), 3.94 (s, 2H), 2.77 – 2.50 (m, 4H), 1.90 – 1.74 (m, 4H), 1.25 (s, 9H). HRMS (ESI): m/z calculated for C_26_H_32_N_2_O_2_ + H^+^ [M + H^+^]: 405.2542; Found: 405.2535.

#### 5-(((4-methoxybenzyl)oxy)methyl)-7-(pyrrolidin-1-ylmethyl)quinolin-8-ol(4i)

^1^H NMR (500 MHz, Chloroform-*d*) δ 8.89 (dd, *J* = 4.1, 1.7 Hz, 1H), 8.38 (dd, *J* = 8.5, 1.7 Hz, 1H), 7.41 (dd, *J* = 8.5, 4.1 Hz, 1H), 7.30 – 7.27 (m, 2H), 7.25 (s, 1H), 6.97 – 6.83 (m, 2H), 4.83 (s, 2H), 4.52 (s, 2H), 4.01 (s, 2H), 3.82 (s, 3H), 2.86 – 2.62 (m, 4H), 1.96 – 1.79 (m, 4H). HRMS (ESI): m/z calculated for C_23_H_26_N_2_O_3_ + H^+^ [M + H^+^]: 379.2022; Found: 379.2025.

#### 5-(((4-(benzyloxy)benzyl)oxy)methyl)-7-(pyrrolidin-1-ylmethyl)quinolin-8-ol(4j)

^1^H NMR (400 MHz, Chloroform-*d*) δ 8.80 (dd, *J* = 4.1, 1.6 Hz, 1H), 8.30 (dd, *J* = 8.5, 1.7 Hz, 1H), 7.39–7.29 (m, 5H), 7.28–7.20 (m, 4H), 6.94–6.85 (m, 2H), 5.00 (s, 2H), 4.75 (s, 2H), 4.44 (s, 2H), 3.97 (s, 2H), 2.80–2.60 (m, 4H), 1.90–1.76 (m, 4H). HRMS (ESI): m/z calculated for C_29_H_30_N_2_O_3_ + H^+^ [M + H^+^]: 455.2335; Found: 455.2335.

#### N-(4-(((8-hydroxy-7-(pyrrolidin-1-ylmethyl)quinolin-5-yl)methoxy) methyl)phenyl)acetamide(4k)

^1^H NMR (400 MHz, Chloroform-*d*) δ 8.81 (dd, *J* = 4.1, 1.7 Hz, 1H), 8.30 (dd, *J* = 8.5, 1.7 Hz, 1H), 7.42 (d, *J* = 8.4 Hz, 2H), 7.34 (dd, *J* = 8.5, 4.1 Hz, 1H), 7.25–7.20 (m, 2H), 7.18 (s, 1H), 4.75 (s, 2H), 4.46 (s, 2H), 3.94 (s, 2H), 2.76–2.66 (m, 4H), 2.11 (s, 3H), 1.88–1.76 (m, 4H). HRMS (ESI): m/z calculated for C_24_H_27_N_3_O_3_ + H^+^ [M + H^+^]: 406.2131; Found: 406.2136.

#### 7-(pyrrolidin-1-ylmethyl)-5-(((3,4,5-trimethoxybenzyl)oxy)methyl)quinolin-8-ol(4l)

^1^H NMR (400 MHz, Chloroform-*d*) δ 8.82 (dd, *J* = 4.1, 1.6 Hz, 1H), 8.34 (dd, *J* = 8.5, 1.7 Hz, 1H), 7.34 (dd, *J* = 8.5, 4.1 Hz, 1H), 7.19 (s, 1H), 6.48 (s, 2H), 4.79 (s, 2H), 4.42 (s, 2H), 3.93 (s, 2H), 3.77 (s, 3H), 3.76 (s, 6H), 2.78 – 2.48 (m, 4H), 1.89 – 1.66 (m, 4H). HRMS (ESI): m/z calculated for C_25_H_30_N_2_O_5_ + H^+^ [M + H^+^]: 439.2233; Found: 439.2245.

#### General Procure for the synthesis of compounds (5a-5g)

Method A (for **5a-5d**): To a solution of alcohols (6 mmol) in anhydrous THF(30ml) was added sodium hydride (60% dispersion in mineral oil, 0.72g, 18 mmol) at 0 oC. The suspension was stirred at 0 oC for 30 minutes. Salt **2** (1.15g, 5mmol) was added to the suspension. The mixture was stirred at r.t for 3 hours. Water was added to the suspension, the mixture became homogeneous. The mixture was extracted by ethyl acetate and washed with brine, dried over anhydrous sodium sulfate and concentrated to get the crude. The crude compound was purified by flash chromatography (ethyl acetate: hexane 3:1).

Method B(for **5e-5g**): 5-chloromethyl-8-quinolinol hydrochloride **2** (1.15 g, 5 mmol) was suspended in different alcohols (25 mmol) and the mixture was heated at 90°C for 2 h, during which time the solution became completely homogeneous. The solution was then poured to 200 ml diethyl ether. The corresponding hydrochloride salt was recovered by filtration as a bright yellow solid. The yellow solid was then suspended in diethyl ether and aqueous NH_4_OH (2.5% v/v, 7.5 mL, ~1.2 equiv.) was added drop by drop under vigorous stirring until the aqueous phase was basic (pH 8–10). The organic phase was washed with water, dried with anhydrous sodium sulfate and evaporated to dryness to afford the desired products.

#### 5-((furan-2-ylmethoxy)methyl)quinolin-8-ol(5a)

^1^H NMR (400 MHz, Chloroform-*d*) δ 8.72 (dd, *J* = 4.3, 1.6 Hz, 1H), 8.37 (dd, *J* = 8.5, 1.5 Hz, 1H), 7.41 (dd, *J* = 8.5, 4.2 Hz, 1H), 7.38–7.33 (m, 2H), 7.03 (d, *J* = 7.7 Hz, 1H), 6.32–6.24 (m, 2H), 4.79 (s, 2H), 4.42 (s, 2H).

#### 5-((thiophen-2-ylmethoxy)methyl)quinolin-8-ol(5b)

^1^H NMR (400 MHz, Chloroform-*d*) δ 8.80 (dd, *J* = 4.2, 1.6 Hz, 1H), 8.48 (dd, *J* = 8.5, 1.6 Hz, 1H), 7.48 (dd, *J* = 8.5, 4.2 Hz, 1H), 7.42 (d, *J* = 7.7 Hz, 1H), 7.31 (dd, *J* = 4.8, 1.5 Hz, 1H), 7.10 (d, *J* = 7.7 Hz, 1H), 7.00–6.97 (m, 2H), 4.88 (s, 2H), 4.70 (s, 2H).

#### 5-((pyridin-2-ylmethoxy)methyl)quinolin-8-ol(5c)

^1^H NMR (400 MHz, Chloroform-*d*) δ 8.80 (dd, *J* = 4.2, 1.6 Hz, 1H), 8.58–8.52 (m, 2H), 7.67 (td, *J* = 7.7, 1.8 Hz, 1H), 7.50 (dd, *J* = 8.5, 4.2 Hz, 1H), 7.46 (d, *J* = 7.7 Hz, 1H), 7.40 (dt, *J* = 7.8, 1.0 Hz, 1H), 7.21–7.16 (m, 1H), 7.11 (d, *J* = 7.7 Hz, 1H), 4.98 (s, 2H), 4.70 (s, 2H).

#### 5-((cyclohexylmethoxy)methyl)quinolin-8-ol(5d)

^1^H NMR (400 MHz, Chloroform-*d*) δ 8.72 (dd, *J* = 4.2, 1.6 Hz, 1H), 8.43 (dd, *J* = 8.5, 1.6 Hz, 1H), 7.42 (dd, *J* = 8.5, 4.2 Hz, 1H), 7.34 (d, *J* = 7.7 Hz, 1H), 7.02 (d, *J* = 7.7 Hz, 1H), 4.73 (s, 2H), 3.21 (d, *J* = 6.5 Hz, 2H), 1.79 – 1.41 (m, H), 1.23 – 0.97 (m, 3H), 0.91 – 0.73 (m, 2H).

#### 5-(ethoxymethyl)quinolin-8-ol(5e)

^1^H NMR (400 MHz, Chloroform-*d*) δ 8.73 (dd, *J* = 4.2, 1.6 Hz, 1H), 8.43 (dd, *J* = 8.5, 1.6 Hz, 1H), 7.42 (dd, *J* = 8.5, 4.2 Hz, 1H), 7.35 (d, *J* = 7.6 Hz, 1H), 7.03 (d, *J* = 7.7 Hz, 1H), 4.76 (s, 2H), 3.50 (q, *J* = 7.0 Hz, 2H), 1.16 (t, *J* = 7.0 Hz, 3H).

#### 5-(propoxymethyl)quinolin-8-ol(5f)

^1^H NMR (400 MHz, Chloroform-*d*) δ 8.72 (dd, *J* = 4.1, 1.6 Hz, 1H), 8.44 (dd, *J* = 8.5, 1.6 Hz, 1H), 7.42 (dd, *J* = 8.6, 4.2 Hz, 1H), 7.34 (d, *J* = 7.7 Hz, 1H), 7.02 (d, *J* = 7.7 Hz, 1H), 4.75 (s, 2H), 3.38 (t, *J* = 6.7 Hz, 2H), 1.55 (dtd, *J* = 14.0, 7.4, 6.7 Hz, 2H), 0.83 (t, *J* = 7.4 Hz, 3H).

#### 5-(butoxymethyl)quinolin-8-ol(5g)

^1^H NMR (400 MHz, Chloroform-*d*) δ 8.72 (dd, *J* = 4.2, 1.6 Hz, 1H), 8.43 (dd, *J* = 8.5, 1.6 Hz, 1H), 7.42 (dd, *J* = 8.6, 4.2 Hz, 1H), 7.34 (d, *J* = 7.7 Hz, 1H), 7.02 (d, *J* = 7.7 Hz, 1H), 4.75 (s, 2H), 3.42 (t, *J* = 6.6 Hz, 2H), 1.51 (ddt, *J* = 8.9, 7.9, 6.4 Hz, 2H), 1.34 – 1.22 (m, 2H), 0.81 (t, *J* = 7.4 Hz, 3H).

#### General Procure for the preparation of compounds (6a-6g)

An equal molar mixture of the substrates **5**, paraformaldehyde, and the pyrrolidine in anhydrous ethanol (30 mL) was refluxed for 4 hours under argon. After cooling, the solvent was evaporated under reduced pressure. The crude compound was purified by flash chromatography (Dichloromethane: methanol 20: 1) to generate pure products.

#### 5-((furan-2-ylmethoxy)methyl)-7-(pyrrolidin-1-ylmethyl)quinolin-8-ol(6a)

^1^H NMR (400 MHz, Chloroform-*d*) δ 8.87 (dd, *J* = 4.1, 1.6 Hz, 1H), 8.33 (dd, *J* = 8.5, 1.6 Hz, 1H), 7.44 (dd, *J* = 1.9, 0.9 Hz, 1H), 7.40 (dd, *J* = 8.5, 4.1 Hz, 1H), 7.25 (s, 1H), 4.83 (s, 2H), 4.52 (s, 2H), 4.00 (s, 2H), 2.78–2.68 (m, 4H), 1.95 – 1.79 (m, 4H). HRMS (ESI): m/z calculated for C_20_H_22_N_2_O_3_ + H^+^ [M + H^+^]: 339.1709; Found: 339.1711.

#### 7-(pyrrolidin-1-ylmethyl)-5-((thiophen-2-ylmethoxy)methyl)quinolin-8-ol(6b)

^1^H NMR (400 MHz, Chloroform-*d*) δ 8.88 (dd, *J* = 4.1, 1.6 Hz, 1H), 8.37 (dd, *J* = 8.5, 1.7 Hz, 1H), 7.40 (dd, *J* = 8.5, 4.1 Hz, 1H), 7.31 (dd, *J* = 4.9, 1.3 Hz, 1H), 7.22 (s, 1H), 7.04 – 6.96 (m, 2H), 4.85 (s, 2H), 4.73 (s, 2H), 3.99 (s, 2H), 2.81 – 2.56 (m, 4H), 1.99 – 1.74 (m, 4H). HRMS (ESI): m/z calculated for C_20_H_22_N_2_O_2_S + H^+^ [M + H^+^]: 355.1480; Found: 355.1473.

#### 5-((pyridin-2-ylmethoxy)methyl)-7-(pyrrolidin-1-ylmethyl)quinolin-8-ol(6c)

^1^H NMR (400 MHz, Chloroform-*d*) δ 8.89 (dd, *J* = 4.2, 1.6 Hz, 1H), 8.56 (ddd, *J* = 4.9, 1.8, 0.9 Hz, 1H), 8.46 (dd, *J* = 8.5, 1.6 Hz, 1H), 7.67 (td, *J* = 7.7, 1.8 Hz, 1H), 7.45 – 7.39 (m, 2H), 7.29 (s, 1H), 7.18 (ddd, *J* = 7.6, 4.9, 1.2 Hz, 1H), 4.96 (s, 2H), 4.71 (s, 2H), 4.02 (s, 2H), 2.82 – 2.66 (m, 4H), 1.95 – 1.84 (m, 4H). HRMS (ESI): m/z calculated for C_21_H_23_N_3_O_2_ + H^+^ [M + H^+^]: 350.1869; Found: 350.1859.

#### 5-((cyclohexylmethoxy)methyl)-7-(pyrrolidin-1-ylmethyl)quinolin-8-ol(6d)

^1^H NMR (400 MHz, Chloroform-*d*) δ 8.80 (dd, *J* = 4.1, 1.7 Hz, 1H), 8.34 (dd, *J* = 8.5, 1.7 Hz, 1H), 7.36 (dd, *J* = 8.5, 4.1 Hz, 1H), 7.24 (s, 1H), 4.71 (s, 2H), 3.98 (s, 2H), 3.24 (d, *J* = 6.5 Hz, 2H), 2.80 – 2.65 (m, 4H), 1.92 – 1.78 (m, 4H), 1.74 – 1.44 (m, 6H), 1.25 – 1.00 (m, 3H), 0.90 – 0.70 (m, 2H). HRMS (ESI): m/z calculated for C_22_H_30_N_2_O_2_ + H^+^ [M + H^+^]: 355.2386; Found: 355.2385.

#### 5-(ethoxymethyl)-7-(pyrrolidin-1-ylmethyl)quinolin-8-ol(6e)

^1^H NMR (400 MHz, Chloroform-*d*) δ 8.90 (dd, *J* = 4.2, 1.6 Hz, 1H), 8.43 (dd, *J* = 8.5, 1.7 Hz, 1H), 7.45 (dd, *J* = 8.5, 4.1 Hz, 1H), 7.29 (d, *J* = 1.2 Hz, 1H), 4.82 (s, 2H), 4.03 (s, 2H), 3.62 (q, *J* = 7.0 Hz, 2H), 2.88 – 2.61 (m, 4H), 2.04 – 1.73 (m, 4H), 1.27 (t, *J* = 7.0 Hz, 3H). HRMS (ESI): m/z calculated for C_17_H_22_N_2_O_2_ + H^+^ [M + H^+^]: 287.1760; Found: 287.1753.

#### 5-(propoxymethyl)-7-(pyrrolidin-1-ylmethyl)quinolin-8-ol(6f)

^1^H NMR (400 MHz, Chloroform-*d*) δ 8.81 (dd, *J* = 4.2, 1.6 Hz, 1H), 8.34 (dd, *J* = 8.5, 1.7 Hz, 1H), 7.35 (dd, *J* = 8.5, 4.1 Hz, 1H), 7.19 (s, 1H), 4.72 (s, 2H), 3.94 (s, 2H), 3.41 (t, *J* = 6.7 Hz, 2H), 2.75 – 2.58 (m, 4H), 1.87 – 1.76 (m, 4H), 1.56 (dtd, *J* = 14.0, 7.4, 6.6 Hz, 2H), 0.85 (t, *J* = 7.4 Hz, 3H). HRMS (ESI): m/z calculated for C_18_H_24_N_2_O_2_ + H^+^ [M + H^+^]: 315.2073; Found: 315.2059.

#### 5-(butoxymethyl)-7-(pyrrolidin-1-ylmethyl)quinolin-8-ol(6g)

^1^H NMR (400 MHz, Chloroform-*d*) δ 8.81 (dd, *J* = 4.1, 1.6 Hz, 1H), 8.34 (dd, *J* = 8.5, 1.7 Hz, 1H), 7.35 (dd, *J* = 8.5, 4.1 Hz, 1H), 7.20 (s, 1H), 4.72 (s, 2H), 3.95 (s, 2H), 3.45 (t, *J* = 6.6 Hz, 2H), 2.74 – 2.58 (m, 4H), 1.90 – 1.73 (m, 4H), 1.52 (ddt, *J* = 8.9, 7.9, 6.4 Hz, 2H), 1.36 – 1.23 (m, 2H), 0.82 (t, *J* = 7.4 Hz, 3H). HRMS (ESI): m/z calculated for C_19_H_26_N_2_O_2_ + H^+^ [M + H^+^]: 301.1916; Found: 301.1906.

#### General Procure for the preparation of compounds (8a-8b)

To a solution of compound **7** (1 mmol) was added paraformaldehyde (1 mmol) and piperidine (1 mmol for **8a**) or morpholine (1 mmol for **8b**). The mixture was stirred under reflux for 4 hours. The solvent was then evaporated to get the crude product. The crude product was purified by flash chromatography (Dichloromethane: methanol 20: 1).

#### 5-((benzyloxy)methyl)-7-(piperidin-1-ylmethyl)quinolin-8-ol(8a)

^1^H NMR (400 MHz, Chloroform-*d*) δ 8.82 (dd, *J* = 4.1, 1.7 Hz, 1H), 8.29 (dd, *J* = 8.5, 1.7 Hz, 1H), 7.32 (dd, *J* = 8.5, 4.2 Hz, 1H), 7.29–7.20 (m, 5H), 7.09 (s, 1H), 4.76 (s, 2H), 4.49 (s, 2H), 3.77 (s, 2H), 2.52 (s, 4H), 1.61 (p, *J* = 5.6 Hz, 4H), 1.44 (s, 2H). HRMS (ESI): m/z calculated for C_22_H_24_N_2_O_3_ + H^+^ [M + H^+^]: 363.2073; Found: 363.2080.

#### 5-((benzyloxy)methyl)-7-(morpholinomethyl)quinolin-8-ol(8b)

^1^H NMR (400 MHz, Chloroform-*d*) δ 8.88 (dd, *J* = 4.1, 1.6 Hz, 1H), 8.40 (dd, *J* = 8.5, 1.6 Hz, 1H), 7.43 (dd, *J* = 8.5, 4.2 Hz, 1H), 7.38–7.29 (m, 5H), 7.28 (s, 1H), 4.85 (s, 2H), 4.58 (s, 2H), 3.86 (s, 2H), 3.81–3.75 (m, 4H), 2.70–2.55 (m, 4H). HRMS (ESI): m/z calculated for C_23_H_26_N_2_O_2_ + H^+^ [M + H^+^]: 365.1865; Found: 365.1850.

#### General Procure for the preparation of compounds (9a-9b)

The method to make compounds **9a-9b** was the same as the way to make compounds **5e- 5g**.

#### 5-(phenethoxymethyl)quinolin-8-ol(9a)

^1^H NMR (400 MHz, Chloroform-*d*) δ 8.77 (dd, *J* = 4.2, 1.6 Hz, 1H), 8.30 (dd, *J* = 8.5, 1.5 Hz, 1H), 7.40–7.35 (m, 2H), 7.28–7.14 (m, 5H), 7.07 (d, *J* = 7.7 Hz, 1H), 4.82 (s, 2H), 3.72 (t, *J* = 7.0 Hz, 2H), 2.90 (t, *J* = 7.0 Hz, 2H).

#### 5-((3-phenylpropoxy)methyl)quinolin-8-ol(9b)

^1^H NMR (400 MHz, Chloroform-*d*) δ 8.80 (dd, *J* = 4.2, 1.6 Hz, 1H), 8.51 (dd, *J* = 8.5, 1.6 Hz, 1H), 7.50 (dd, *J* = 8.5, 4.2 Hz, 1H), 7.41 (d, *J* = 7.7 Hz, 1H), 7.25 – 7.22 (m, 2H), 7.20 – 7.14 (m, 1H), 7.13 – 7.08 (m, 3H), 4.83 (s, 2H), 3.50 (t, *J* = 6.3 Hz, 2H), 2.65 (t, *J* = 8.6 Hz, 2H), 1.97 – 1.86 (m, 2H).

#### General Procure for the preparation of compounds (10a-10b)

An equimolar mixture of the substrates **9**, paraformaldehyde, and the pyrrolidine in anhydrous ethanol (30 mL) was refluxed for 4 hours under argon. After cooling, the solvent was evaporated under reduced pressure. The crude product was purified by flash chromatography (Dichloromethane: methanol 20: 1) to generate pure products.

#### 5-(phenethoxymethyl)-7-(pyrrolidin-1-ylmethyl)quinolin-8-ol(10a)

^1^H NMR (400 MHz, Chloroform-*d*) δ 8.86 (dd, *J* = 4.2, 1.7 Hz, 1H), 8.23 (dd, *J* = 8.5, 1.7 Hz, 1H), 7.32 (dd, *J* = 8.5, 4.2 Hz, 1H), 7.27 (d, *J* = 2.4 Hz, 1H), 7.26–7.16 (m, 5H), 4.80 (s, 2H), 3.98 (s, 2H), 3.74 (t, *J* = 7.0 Hz, 2H), 2.91 (t, *J* = 7.0 Hz, 2H), 2.75–2.65 (m, 4H), 1.95–1.80 (m, 4H). HRMS (ESI): m/z calculated for C_23_H_26_N_2_O_2_ + H^+^ [M + H^+^]: 363.2073; Found: 363.2060.

#### 5-((3-phenylpropoxy)methyl)-7-(pyrrolidin-1-ylmethyl)quinolin-8-ol(10b)

^1^H NMR (400 MHz, Chloroform-*d*) δ 8.89 (dd, *J* = 4.1, 1.7 Hz, 1H), 8.42 (dd, *J* = 8.5, 1.6 Hz, 1H), 7.43 (dd, *J* = 8.5, 4.1 Hz, 1H), 7.26 – 7.19 (m, 3H), 7.19 – 7.14 (m, 1H), 7.14 – 7.10 (m, 2H), 4.80 (s, 2H), 3.99 (s, 2H), 3.53 (t, *J* = 6.4 Hz, 2H), 2.75 – 2.64 (m, 6H), 1.99 – 1.83 (m, 6H).

HRMS (ESI): m/z calculated for C_24_H_28_N_2_O_2_ + H^+^ [M + H^+^]: 377.2229; Found: 377.2238.

#### General Procure for the preparation of compounds (11a-11b)

To a solution of benzyl mercaptan (6 mmol for **12a**) or N-benzymethylamine (6 mmol for **12b**) in anhydrous THF(30ml) was added sodium hydride (60% dispersion in mineral oil, 0.72g, 18 mmol) at 0 oC. The suspension was stirred at 0 oC for 30 minutes. Salt **2** (1.15g, 5mmol) was added to the suspension. The mixture was stirred at r.t for 3 hours. Water was added to the suspension, the mixture became homogeneous. The mixture was extracted by ethyl acetate and washed with brine, dried over anhydrous sodium sulfate and concentrated to get the crude. The crude product was purified by flash chromatography (ethyl acetate: hexane 3:1).

#### 5-((benzylthio)methyl)-7-(pyrrolidin-1-ylmethyl)quinolin-8-ol(11a)

^1^H NMR (400 MHz, Chloroform-*d*) δ 8.78 (dd, *J* = 4.2, 1.6 Hz, 1H), 8.30 (dd, *J* = 8.6, 1.5 Hz, 1H), 7.44 (dd, *J* = 8.5, 4.2 Hz, 1H), 7.37–7.26 (m, 5H), 7.06 (d, *J* = 7.8 Hz, 1H), 3.96 (s, 2H), 3.65 (s, 2H).

#### 5-((benzyl(methyl)amino)methyl)quinolin-8-ol(11b)

^1^H NMR (400 MHz, Chloroform-*d*) δ 8.82 (dd, *J* = 4.2, 1.6 Hz, 1H), 8.62 (dd, *J* = 8.5, 1.6 Hz, 1H), 7.47 (dd, *J* = 8.6, 4.2 Hz, 1H), 7.40 (d, *J* = 7.7 Hz, 1H), 7.37–7.32 (m, 4H), 7.32–7.26 (m, 1H), 7.13 (d, *J* = 7.7 Hz, 1H), 3.85 (s, 2H), 3.58 (s, 2H), 2.20 (s, 3H).

#### General Procure for the preparation of compounds (12a-12b)

To a solution of compound **11** (1 mmol) was added paraformaldehyde (1 mmol) and pyrrolidine (1mmol). The mixture was stirred under reflux for 4 hours. The solvent was then evaporated to get the crude product. The crude product was purified by flash chromatography (Dichloromethane: methanol 20: 1) to get a light yellow solid.

#### 5-((benzylthio)methyl)-7-(pyrrolidin-1-ylmethyl)quinolin-8-ol(12a)

^1^H NMR (400 MHz, Chloroform-*d*) δ 8.78 (dd, *J* = 4.2, 1.6 Hz, 1H), 8.17 (dd, *J* = 8.6, 1.6 Hz, 1H), 7.32 (dd, *J* = 8.6, 4.1 Hz, 1H), 7.28 – 7.15 (m, 5H), 7.14 (s, 1H), 3.97 (s, 2H), 3.89 (s, 2H), 3.60 (s, 2H), 2.82 – 2.65 (m, 4H), 1.90– 1.80 (m, 4H). HRMS (ESI): m/z calculated for C_22_H_24_N_2_OS + H^+^ [M + H^+^]: 365.1688; Found: 365.1689.

#### 5-((benzyl(methyl)amino)methyl)-7-(pyrrolidin-1-ylmethyl)quinolin-8-ol(12b)

^1^H NMR (400 MHz, Chloroform-*d*) δ 8.87 (dd, *J* = 4.1, 1.6 Hz, 1H), 8.57 (dd, *J* = 8.6, 1.7 Hz, 1H), 7.43–7.31 (m, 5H), 7.31–7.27 (m, 1H), 7.24 (s, 1H), 3.93 (s, 2H), 3.91 (s, 2H), 3.68 (s, 2H), 2.60–2.48 (m, 4H), 2.30 (s, 3H), 1.85–1.70 (m, 4H). HRMS (ESI): m/z calculated for C_23_H_27_N_3_O + H^+^ [M + H^+^]: 362.2232; Found: 362.2224.

### Cell culture and cell viability assay

The anti-proliferative effect of UC-112 and its analogs were tested in human melanoma (A375, M14 and M14/LCC6MDR1) and human prostate cancer (PC-3) cell lines. All the cell lines were purchased from ATCC (American Type Culture Collection, Manassas, VA). The cancer cells were cultured using the supplemented cell culture medium as described before [32] at 37°C in a humidified atmosphere containing 5% CO2. 5000 cells in logarithm growing phase were seeded overnight into each well of a 96-well plate. Then the cells were continuously incubated with sequential diluted compound solution (10 nM to 100 μM, 100 μl per well) in cell culture medium. After 48 h treatment, the cell viability was determined in MTS assay and IC_50_ was calculated (*n* = 4) as described before [19, 33].

### NCI-60 screening

Four compounds including the parental compound UC-112 were submitted to National Cancer Institute for its NCI-60 cell line screening, initially tested at one concentration (10 μM), and subsequently selected for full five concentration testing following the standard protocols disclosed by NCI [34]. In brief, cells were plated into 96 well micro-titer plates 24 h prior to the treatment of compound solution for 48 h, then the cell viability was read out through absorbance of sulforhodamine B (SRB) staining.

### Drug-like property profiling for compound 4g

All these experiments were performed by a contract research service company, Eurofins Panlabs Inc. (Study Number 100017964). Aqueous solubility (μM) was determined using the shake-flask method by comparing the peak area of the principal peak in a calibration standard (200 μM) containing organic solvent (methanol/water, 60/40, v/v) with the peak area of the corresponding peak in a buffer sample. Metabolic stability study was carried out by incubating compound **4g** with human liver microsomes. Metabolic stability which is expressed as percent of the parent compound remaining, was calculated by comparing the peak area of the compound at the time point relative to that at time-0. The half-life (T1/2) was estimated from the slope of the initial linear range of the logarithmic curve of compound remaining (%) vs. time. In the cytochrome P450 inhibition study, human liver microsomes (0.1 mg/mL) were incubated with different substrates and compound **4g** for 10 minutes. After the incubation, peak areas corresponding to the metabolite of each substrate were recorded. The percent of control activity was then calculated by comparing the peak area obtained in the presence of compound **4g** to that obtained in the absence of compound **4g**. Subsequently, the percent inhibition was calculated by subtracting the percent control activity from 100.

### Caspase functional assay

The caspase 3/7 activity of cancer cells treated by DMSO control or compound of interest was analyzed using Glo Caspase-Glo 3/7 kit from Promega Corporation (Madison, WI) as per manufacturer’s instructions in similar protocol as described before [19]. The readings of relative luminescence unit were normalized by the cell viability read from the same well determined by compatible CytoTox-Fluor Cytotoxicity assay kit (Promega, WI).

### Western blotting

Lysates of A375 cells treated by the compound solution for 24 h were used to determine the change of IAP protein levels through western blotting. Primary rabbit antibodies against survivin, XIAP, cIAP1, cIAP2, livin and GAPDH were purchased from Cell Signaling Technology, Inc. (Danvers, MA) and used according to manufacture instructions as reported previously[19]. Protein lane intensities were quantified by ImageJ software (US National Institutes of Health, Bethesda, MD).

### Molecular modeling

The molecular docking studies were performed following similar procedure as described before [19, 35]in Schrodinger Molecular Modeling Suite 2014 (Schrodinger Inc., Portland, OR). All the ligands were prepared to generate various conformation before being docked into the SMAC AVPI binding site of a human survivin crystal structure (Protein Data Bank entry: 3UIH). Molecular dynamic calculation was done after the docking to minimize the energy of potential ligand binding poses. Results were visualized using the Maestro interface of the Schrodinger software.

### DARTS assay

Drug affinity responsive target stability (DARTS) assay was performed to identify the protein targets of UC-112 analogs in A375 or M14 cell lysates following the protocols described in the literature [28–31]. Briefly, A375 or M14 lysates were prepared in non-denaturing M-PER lysis buffer (Thermo Fisher Scientific, Waltham, MA) with protease and phosphatase inhibitors. Then TNC (Tris, NaCl, CaCl2) buffer was added into cell lysates before the total protein concentration of lysate being determined by BCA protein assay kit (Thermo Fisher Scientific, Waltham, MA). The lysates were split into two groups: one group was added with compound of interest solution, the other group was added with same amount of vehicle (DMSO). The samples were mixed thoroughly and incubated at room temperature for 1 h. Then 10 mg/mL pronase stock solution was added into both compound treated or vehicle control groups to achieve the final dilution of 1:100, 1:300, 1:1000 and 1:3000. One aliquot of each group was kept as un-digested control. The proteolysis was performed at room temperature for 30 min before ice cold protease inhibitor stock being added into the mixture. Then SDS loading buffer was added into all the samples and heated to 70°C for 10 min. Finally, the results were analyzed by SDS-PAGE and immunoblotting.

### *In vivo* anti-tumor efficacy

3 × 10^6^ A375 cells were implanted into the left-side dorsal flank of each nude mouse (*n* = 5) to establish the human melanoma A375 tumor xenograft model as described previously [19, 36]. Compound **4g** were first dissolved in DMSO then diluted by 2% methylcellulose PBS buffer. The proportion of DMSO in final solution was kept at lower than 5%. Mice body weight and the size of tumor were closely monitored during the 3 week continuous treatment (i.p. injection, one dose per day, five days per week). At the end point of the treatment, mice were sacrificed after anesthesia. A375 tumor tissues were isolated then freshly lysed on ice to check the targeted protein levels through Western blotting.

### TUNEL assay

A375 tumor tissues collected from the *in vivo* efficacy study in the above were fixed in formalin phosphate buffer for one week. Then the tissues were processed to get paraffin embedded sections. TUNEL assay was performed using DeadEnd Fluorometric kit (Promega Corporation, Madison, WI) following manufacturer’s instructions. By the end of the experiment, VECTASHIELD Hard Set mounting medium with DAPI (Vector Lab, Inc., Burlingame, CA) was used to mount the tumor slides and stain the nuclei. The final slides were analyzed immediately under a fluorescence microscope (EVOS FL Cell Imaging System, Thermo Fisher Scientific Inc., NY).

## Conclusion

**Fig 14:**
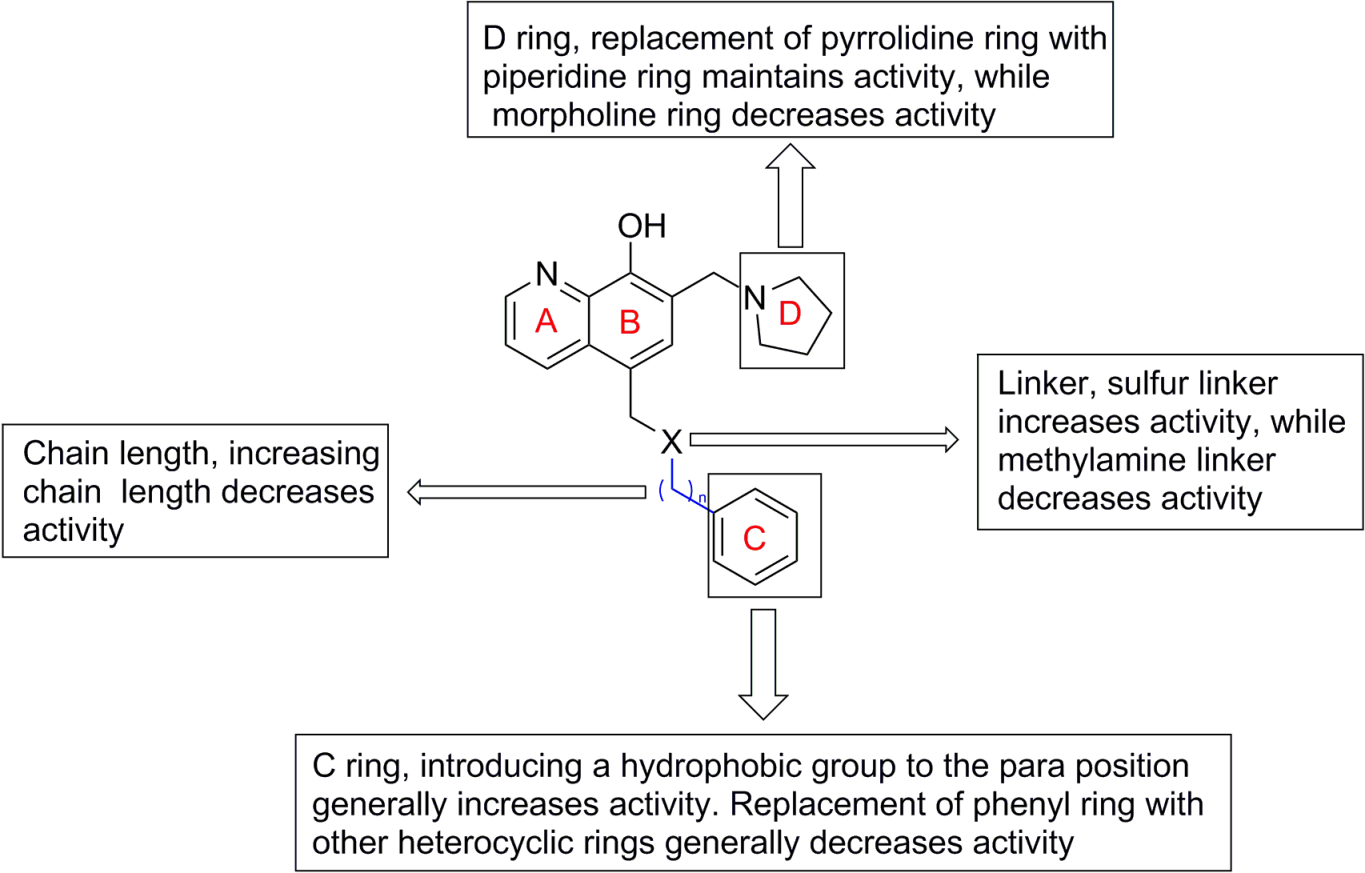
Structure activity relationships of UC-112 analogs.

In summary, a series of novel analogs were designed and synthesized based on targeted structural modification of the lead compound UC-112. Structure-activity relationships (Fig 14) were investigated by making modifications to C ring, D ring and the linker. Several analogs showed excellent anti-proliferative activities and could also effectively overcome Pgp-mediated multiple drug resistance. This scaffold as demonstrated by our most potent compound **4g** has showed good drug-like properties which is important for further development. Preliminary studies of action mechanism confirmed that the new analogs maintain their mode of action by selectively down-regulating the level of survivin as the parent compound UC-112. Compound **4g** effectively inhibited the growth of A375 xenograft tumor *in vivo*. Further optimization of this scaffold and efforts to produce X-ray crystal structures of survivin protein in complex with these novel survivin inhibitors are currently being carried out to generate more potent and selective survivin inhibitors based on this unique platform.

## Supporting Information

S1 Fig Mean graph report for UC-112 (NSC D-782181) from NCI-60 cell line five dose anti-proliferative screening. (TIF)

S2 Fig Mean graph report for compound 12a (NSC D-782184) from NCI-60 cell line five dose anti-proliferative screening. (TIF)

S3 Fig Mean graph report for compound 4c (NSC D-782182) from NCI-60 cell line five dose anti-proliferative screening. (TIF)

S4 Fig Mean graph report for compound 4g (NSC D-782180) from NCI-60 cell line five dose anti-proliferative screening. (TIF)

S5 Fig ImageJ quantification data of western blotting analysis results in Fig 2. (TIF)
