# Supplementary figures and images for: Design, Synthesis and Structure-Activity Relationship Studies of Novel Survivin Inhibitors with Potent Anti-Proliferative Properties
Source: PLoS One. 2015 Jun 12;10(6):e0129807. doi: 10.1371/journal.pone.0129807 (PMC4466525; doi:10.1371/journal.pone.0129807)

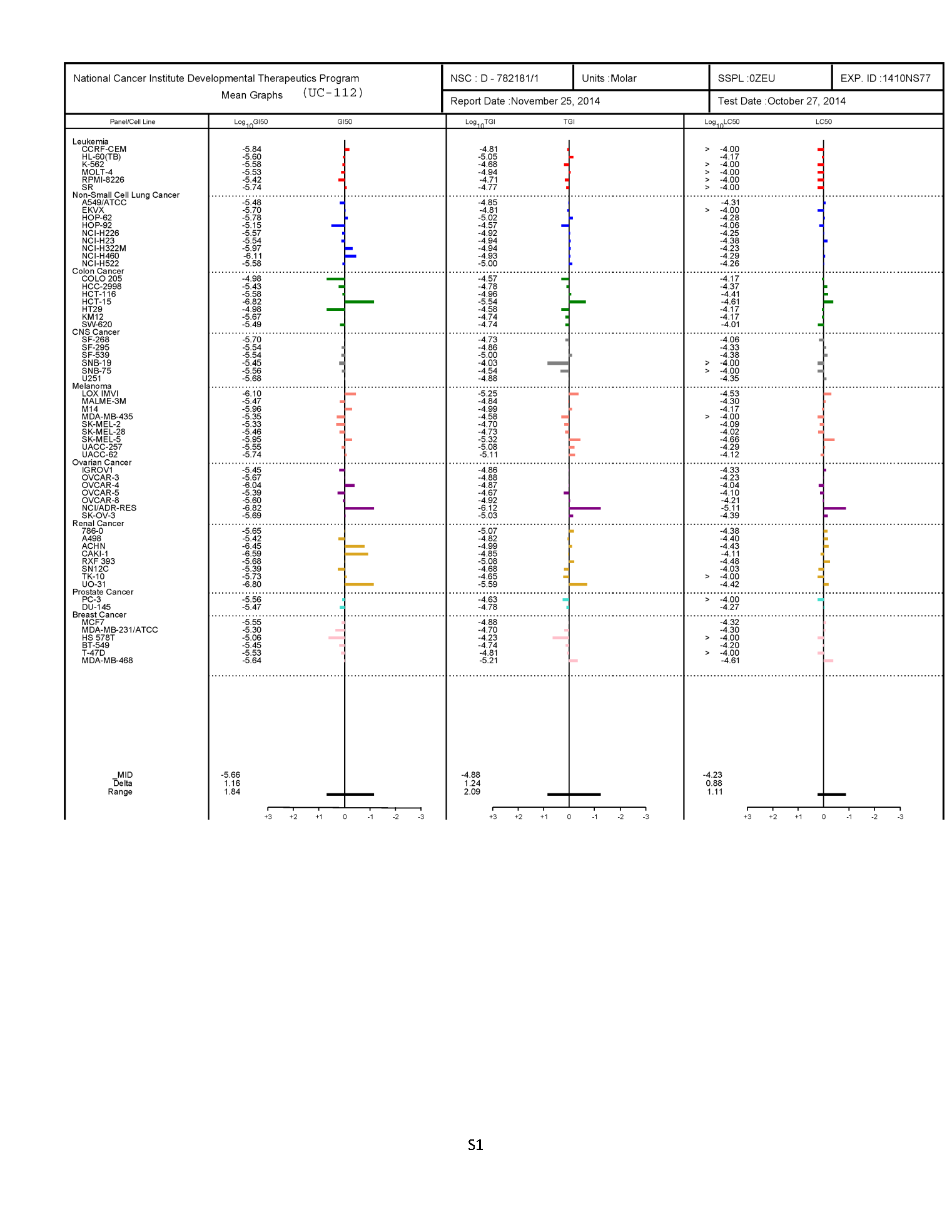

Supplement: S1 Fig — (TIF) [file pone.0129807.s001.tif]

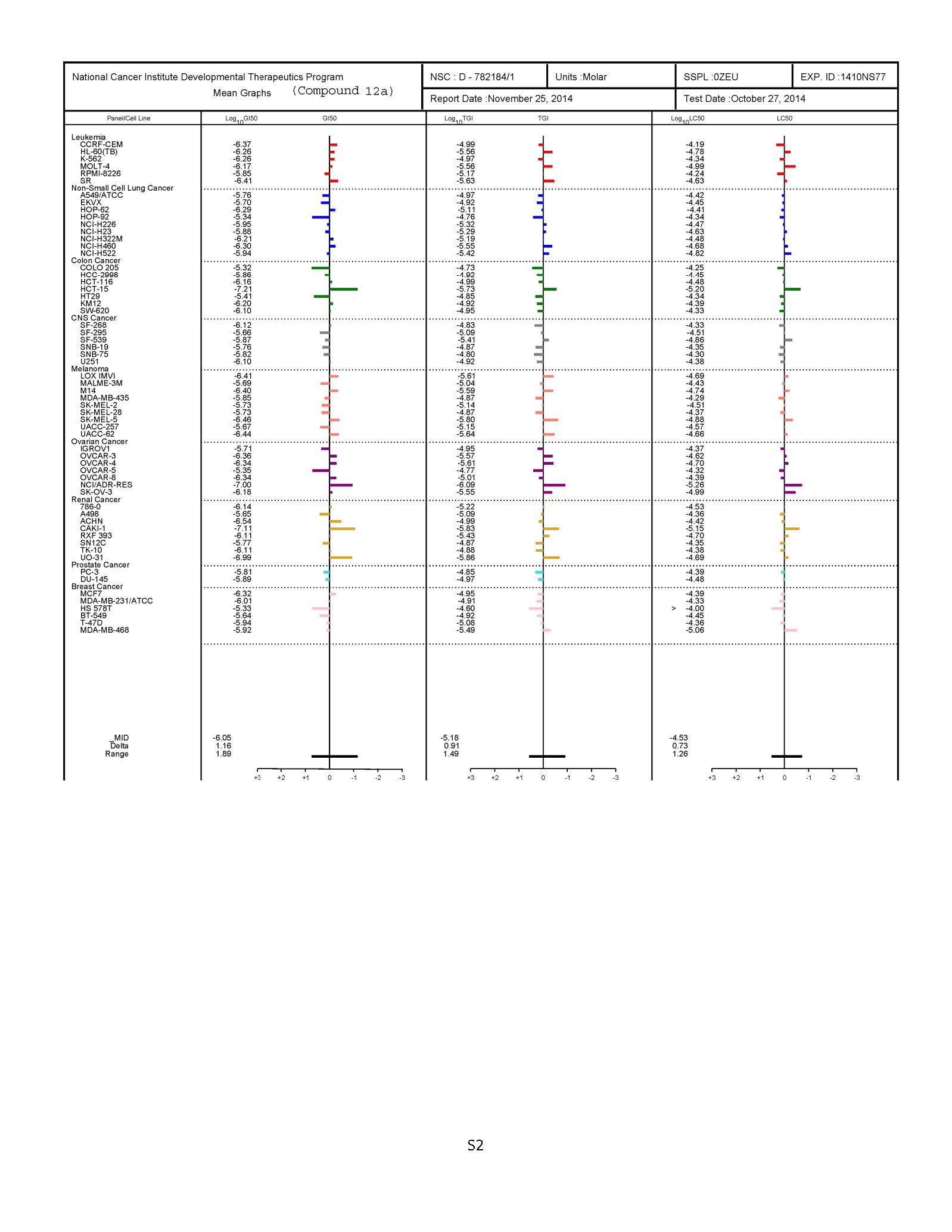

Supplement: S2 Fig — (TIF) [file pone.0129807.s002.tif]

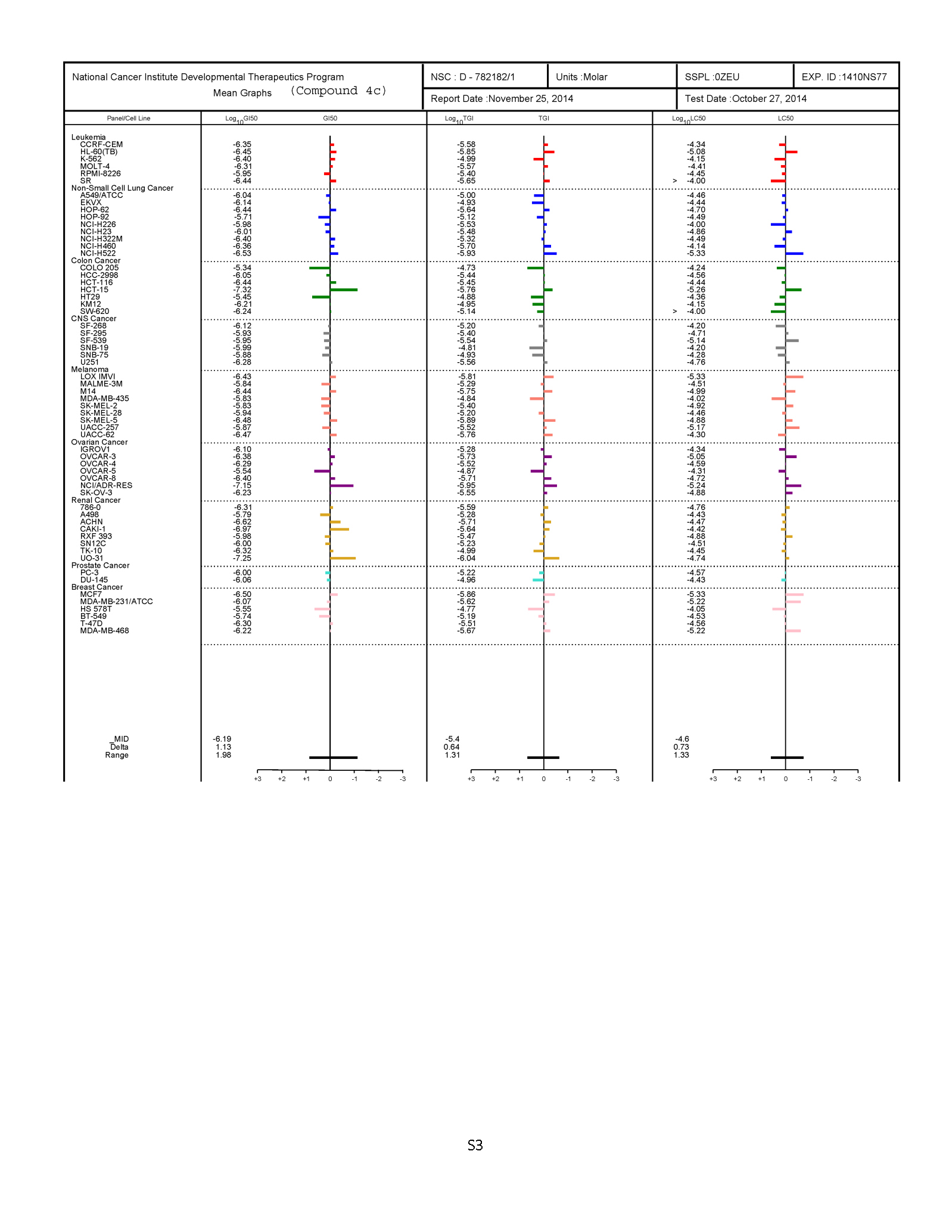

Supplement: S3 Fig — (TIF) [file pone.0129807.s003.tif]

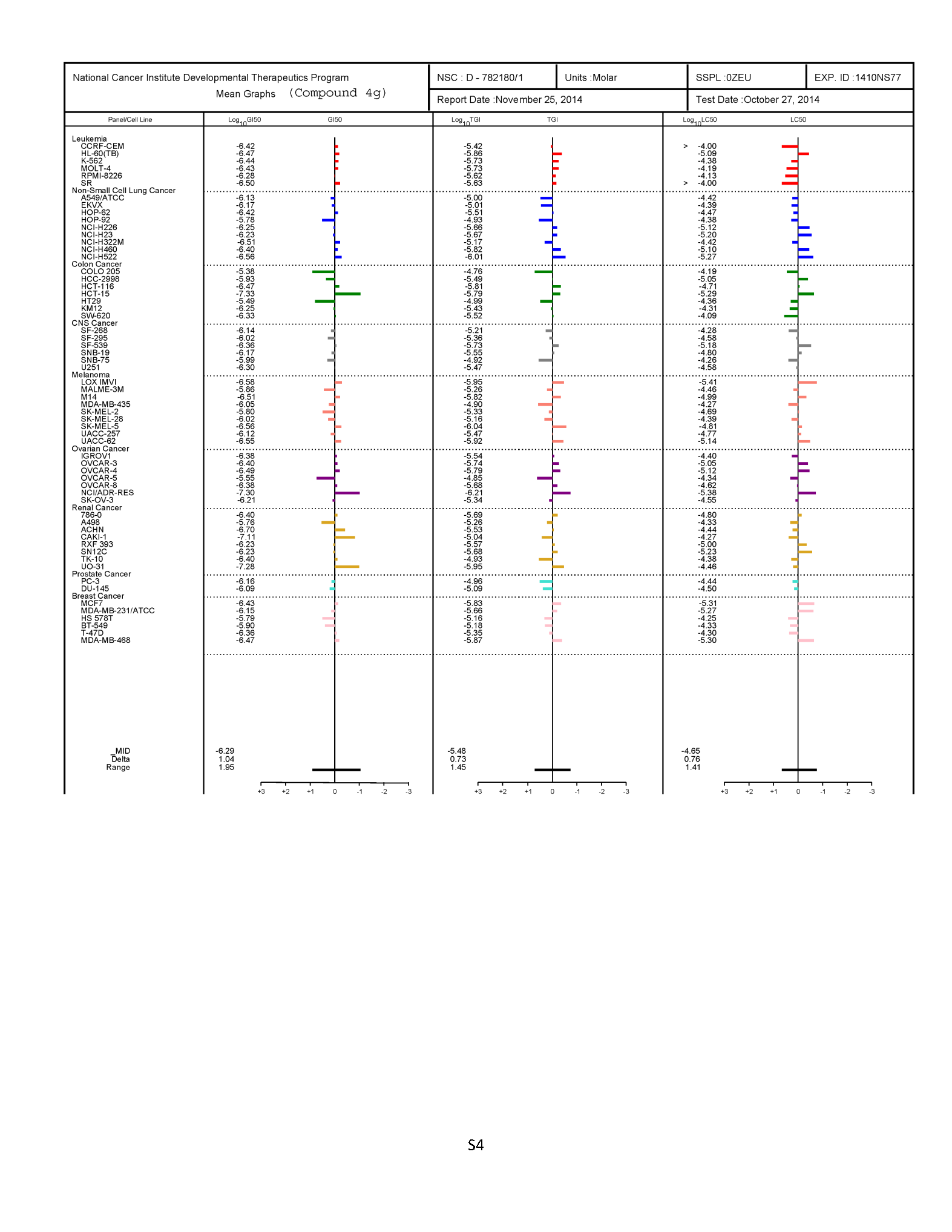

Supplement: S4 Fig — (TIF) [file pone.0129807.s004.tif]

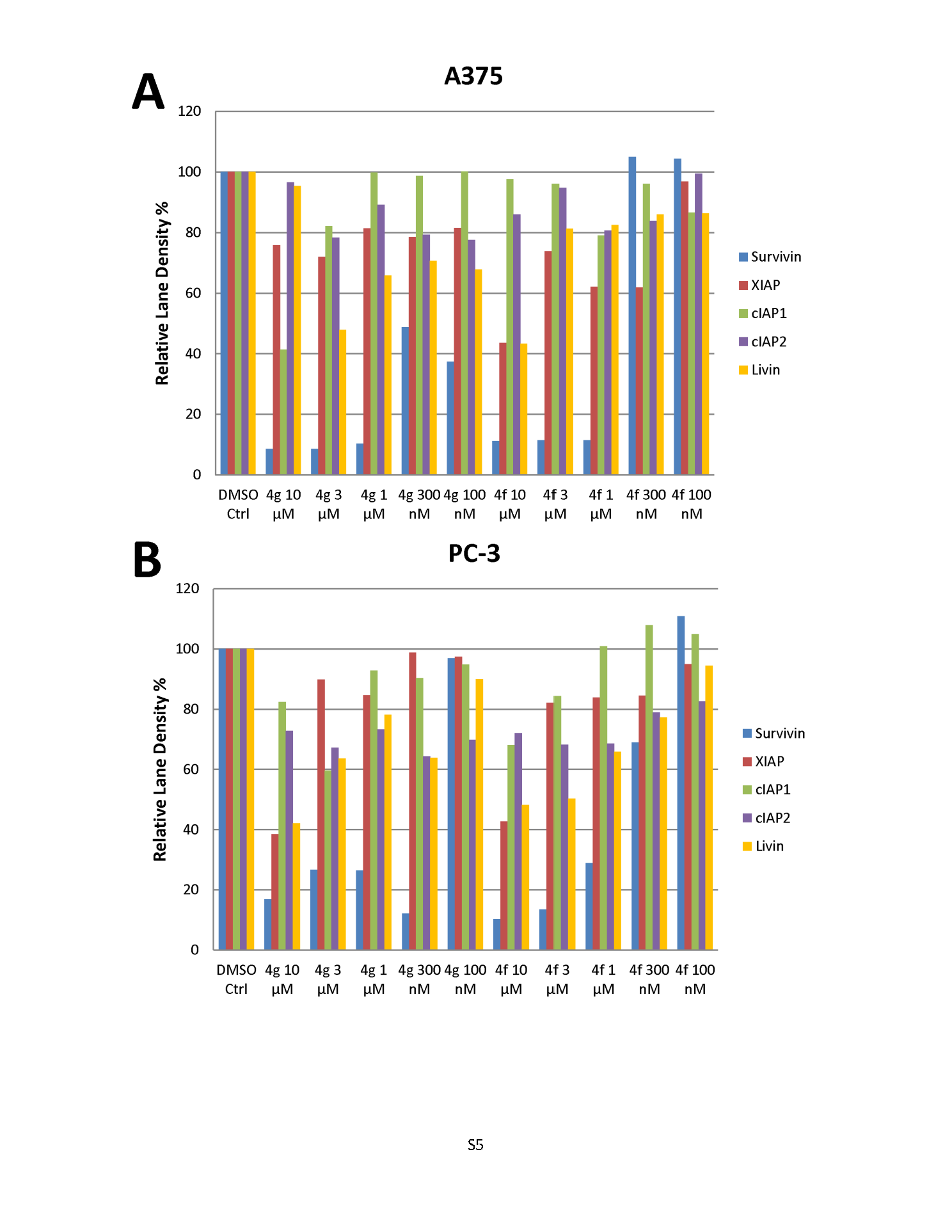

Supplement: S5 Fig — (TIF) [file pone.0129807.s005.tif]
